# PFKP is required for chemoresistant phenotype of breast cancer through modulating the formation of CD133^+^ cancer stem like cells

**DOI:** 10.1186/s43556-026-00454-z

**Published:** 2026-04-23

**Authors:** Kai Fang, Yue Ma, Lihua Li, Yan Yue, Hang Ruan, Sidong Xiong

**Affiliations:** 1 The Fourth Affiliated Hospital of Soochow University, Institutes of Biology and Medical Sciences, Suzhou Medical College of Soochow University, Soochow University, Suzhou, 215123 China; 2 Department of Oncology Institute, The Affiliated Hospital of Jiangnan University, Wuxi, 214000 China; 3 MOE Key Laboratory of Geriatric Diseases and Immunology, Suzhou Medical College of Soochow University, Soochow University, Suzhou, 215123 China; 4 Jiangsu Key Laboratory of Infection and Immunity, Institutes of Biology and Medical Sciences, Soochow University, Suzhou, 215123 China

**Keywords:** Breast cancer, PFKP, Glycolysis, Cancer stem-like cells, Cancer-associated fibroblasts, Chemoresistance

## Abstract

Breast cancer is the foremost cause of cancer-related death in women globally, and taxane-anthracycline (TA) combination regimens represent standard frontline chemotherapy. Although widely administered, the pathological complete response rate to TA therapy is less than 30%, and chemoresistance remains a major barrier to effective disease control, frequently leading to relapse and poor survival. Both metabolic reprogramming and tumor microenvironmental remodeling are closely associated with treatment failure, yet how they interact to drive TA resistance remains largely unclear. Here we show that phosphofructokinase platelet (PFKP), a key glycolytic enzyme, is highly expressed in breast cancer. PFKP drives glycolysis and promotes CD133^+^ cancer stem-like cells (CSLCs) that are inherently TA-resistant. Moreover, PFKP-overexpressing cancer cells stimulate cancer-associated fibroblasts (CAFs), which in turn augment CD133^+^ CSLC formation via the CXCL16/CXCR6 axis, establishing a feedforward loop that reinforces chemoresistance. These results reveal a previously unappreciated mechanism by which a glycolytic enzyme in cancer cells orchestrates stromal crosstalk to sustain a chemotherapy-refractory niche. By identifying PFKP as a key driver and the PFKP–CSLC–CAF axis as an actionable target, our work moves the field beyond the traditional view of metabolic reprogramming as a cell-autonomous event. Disrupting this axis—for instance, by PFKP inhibition or CXCL16/CXCR6 blockade—may restore TA sensitivity in aggressive basal-type breast cancer, offering a promising strategy to improve long-term outcomes for hard-to-treat patients.

**Supplementary Information:**

The online version contains supplementary material available at 10.1186/s43556-026-00454-z.

## Introduction

Breast cancer (BC) remains a significant global health challenge [1]. Taxanes and anthracyclines (TA) constitute standard components of chemotherapy regimens for BC patients [2–4]. Despite their widespread use, fewer than 30% of BC patients achieve a pathological complete response (pCR) following TA-based chemotherapy [5–7]. The mechanisms underlying chemoresistance in BC remain largely unclear.

Intra-tumor heterogeneity significantly contributes to the varied responses of tumor cells to chemotherapy, thereby affecting the overall effectiveness of the treatment [8]. A defining feature of cancers, including BC, is the presence of cancer stem-like cells (CSLCs), which drive tumor initiation, progression, and chemoresistance [9–12]. BC stem-like cells (BCSLCs) are identified by markers such as ALDH^+^, CD24^-^/CD44^+^, and CD133^+^ [13–15]. CSLCs are more resistant to chemotherapy than non-CSLCs [16]. However, the specific BCSLCs subpopulation responsible for resistance to TA-based drugs has not yet been conclusively identified.

The development of chemoresistance is complex, influenced not only by intracellular factors but also by the tumor microenvironment (TME) [17]. Within the TME, cancer-associated fibroblasts (CAFs) and tumor-associated macrophages play particularly significant roles in contributing to TME heterogeneity. Specifically, CAFs are known to induce tumor cells to develop resistance to various anticancer treatments [18]. Moreover, CAFs have been shown to promote the formation of CSLCs in various solid tumors [19]. This dual role of CAFs in both drug resistance and CSLC formation underscores their importance in the complex dynamics of chemoresistance. However, the specific phenotype of BCSLCs induced by CAFs remains to be elucidated.

In this study, we aim to identify the specific phenotype of CSLCs that contribute to BC's resistance to TA-based drugs. We also seek to elucidate the intracellular and extracellular mechanisms involved, thereby laying a theoretical foundation for strategies to improve BC patients' responsiveness to TA-based chemotherapy.

## Results

### Multi-omics data analysis suggests PFKP may enhance chemoresistance in BC

**Fig. 1:**
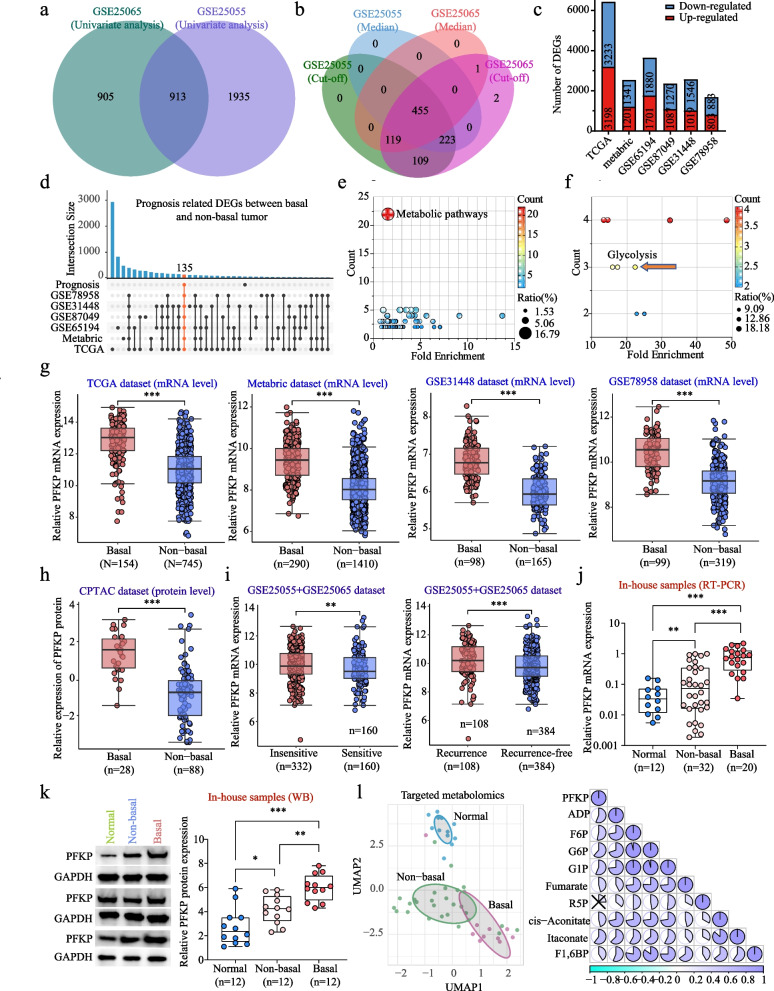
Identification of PFKP as a key regulator of chemoresistance in BC. **a** Univariate analysis identified 913 genes significantly associated with recurrence in BC patients following TA-based chemotherapy (all P < 0.05) in the GSE25055 (n = 308) and GSE25065 (n = 198) datasets. **b** Kaplan–Meier analysis revealed that 455 of 913 genes were significantly linked to relapse-free survival (all P < 0.05). **c** Differential expressed gene (DEG) analysis between basal-type and non-basal-type BC tumor tissues was performed using multiple public datasets (GSE31448, GSE65194, GSE87049, GSE78958, METABRIC, and TCGA). **d** UpSet analysis identified 135 overlapping prognosis-associated genes that were significantly differential expressed between basal-type and non-basal-type BC (all P < 0.05). (e and f) Functional enrichment analysis based on KEGG pathways revealed significant enrichment in metabolic pathways (**e**) and glycolysis-related pathways, including LDHB, FBP1, and PFKP (**f**). **g** and **h** mRNA (**g**) and protein (**h**) expression levels of PFKP were significantly higher in basal-type BC tissues compared to non-basal-type BC. **i** PFKP expression was markedly elevated in chemotherapy-insensitive or recurrent BC patients compared to those who were sensitive or recurrence-free after TA-based chemotherapy. **j** and **k** In-house validation confirmed increased PFKP mRNA (**j**) and protein (**k**) expression in both basal-type and non-basal-type BC tissues compared to adjacent normal tissues, with higher levels observed in basal-type tumors. **l** Targeted quantitative metabolomics revealed metabolic differences among adjacent normal tissues (n=12), non-basal-type breast cancer (BC) tumors (n=28), and basal-type BC tumors (n=10). Specifically, it demonstrated a strong positive correlation between PFKP mRNA expression (**j**) and glycolysis-related metabolites (**l**). Notes: Data are presented as mean ± SD. × represents P > 0.05 and correlation r < 0.2. * represents P < 0.05, ** represents P < 0.01, and *** represents P < 0.001

To identify key molecules linked to chemoresistance, we analyzed datasets GSE25055 (n=308) and GSE25065 (n=198) from BC patients who underwent TA-based chemotherapy. Univariate analysis identified 913 genes significantly associated with recurrence (Fig. 1a). Kaplan-Meier analysis indicated that 455 of these genes were significantly linked to relapse-free survival (Fig. 1b). Patients with basal-type BC exhibited reduced sensitivity to TA-based chemotherapy and a higher risk of recurrence compared to those with non-basal-type BC (Table. S1). We analyzed DEGs between basal-type and non-basal-type BC tumors from multiple datasets (GSE31448, GSE65194, GSE87049, GSE78958, METABRIC, TCGA; Fig. 1c). UpSet analysis revealed that among the 455 prognosis-associated genes, 135 were significantly differentially expressed between subtypes (Fig. 1d). KEGG enrichment analysis showed these 135 genes were significantly enriched in metabolic pathways (Fig. 1e) and glycolytic pathway (LDHB, FBP1, PFKP; Fig. 1f). Phosphofructokinase platelet (PFKP), a critical rate-limiting enzyme in glycolysis, was significantly higher in basal-type BC tumors at both mRNA and protein levels (Fig. 1g-h). Compared to patients with favorable response or remaining recurrence-free after TA-based chemotherapy, those with treatment resistance or recurrence showed markedly increased PFKP expression (Fig. 1i). In our in-house cohort, we similarly observed elevated PFKP expression in basal-type BC tissues compared to non-basal-type tumors, and upregulated expression in non-basal-type BC tissues compared to adjacent normal tissues (Fig. 1j-k). Quantitative metabolomics demonstrated a strong positive correlation between PFKP mRNA expression and glycolysis-related metabolites (Fig. 1l). These findings suggest PFKP may contribute to chemoresistance by activating glycolytic pathways.

### PFKP promotes chemoresistance in BC

**Fig. 2:**
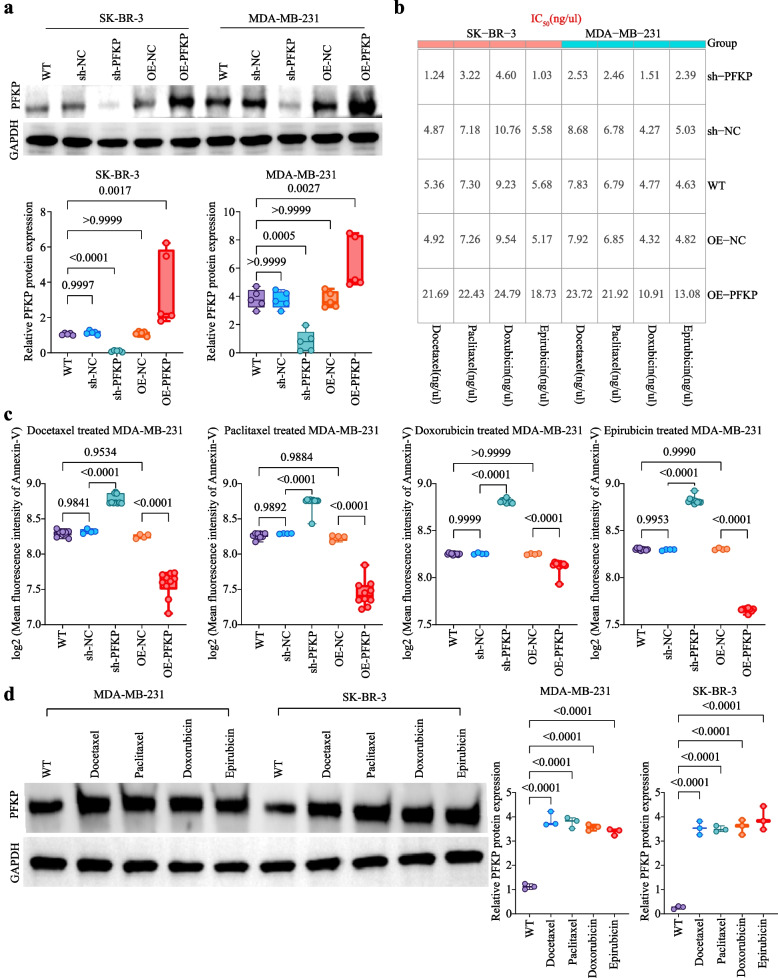
PFKP promotes resistance of BC cells to TA-based drugs *in vitro*. **a** The modulation of PFKP expression in MDA-MB-231 and SK-BR-3 cells was achieved through lentiviral-mediated knockdown or overexpression, and the efficiency of lentiviral transduction was evaluated using WB assay. **b** Cells were treated with various concentrations of docetaxel, paclitaxel, doxorubicin, and epirubicin (0, 0.5, 1, 2, 4, 8, 16, and 32 ng/μL) for 24 h. The CCK-8 assay was then used to detect the IC_50_ values of these TA-based drugs in PFKP-modulated cells. The results showed that IC_50_ values decreased with PFKP silencing, indicating increased drug sensitivity, while IC_50_ values increased with PFKP overexpression, indicating enhanced drug resistance. Each experiment was performed in triplicate. **c** After treating 5 × 10^6^ cells with TA-based drugs (including 5 µg/mL paclitaxel, 5 µg/mL docetaxel, 2.5 µg/mL doxorubicin, or 2.5 µg/mL epirubicin) or without TA-based drugs for 24 h, apoptosis was assessed using Annexin-V staining via flow cytometry in MDA-MB-231 cells. Each experiment was performed at least four times. **d** MDA-MB-231 and SK-BR-3 cells were treated with 5 ng/μL of paclitaxel, docetaxel, doxorubicin, or epirubicin for 24 h. After this initial treatment, the medium was replaced with complete medium. Seventy-two hours later, the cells were re-treated with the same concentrations of drugs for another 24 h. This cycle was repeated ten times. WB analysis showed that compared to the WT group, repeated treatments with paclitaxel, docetaxel, doxorubicin, or epirubicin significantly promoted the expression of PFKP in both MDA-MB-231 and SK-BR-3 cells. Each experiment was performed in triplicate. Notes: Data are presented as mean ± SD

**Fig. 3:**
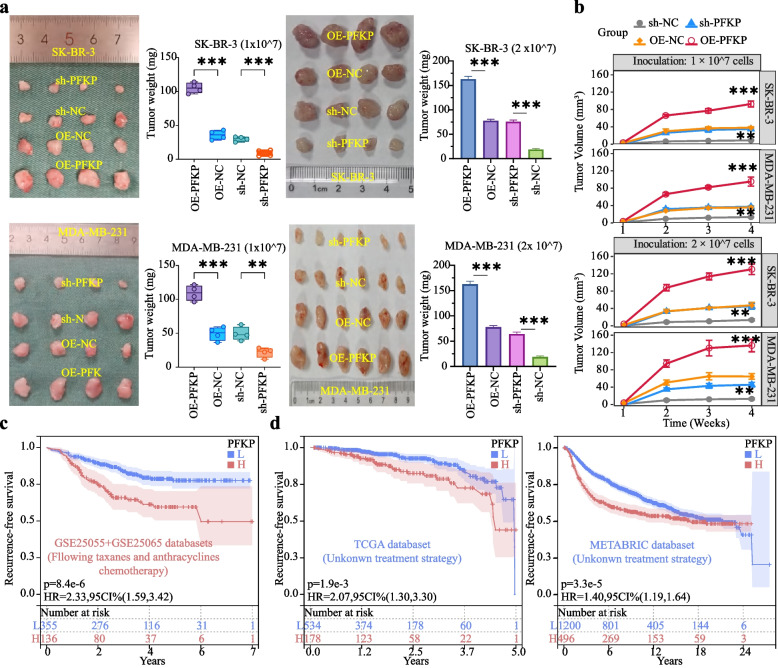
PFKP promotes resistance of BC to TA-based drugs *in vivo*. **a** and **b** A total of 1×10^7^ (n=4) or 2×10^7^ (n=4 or 6) sh-NC, sh-PFKP, OE-NC, and OE-PFKP cells were subcutaneously inoculated into the right flank of nude mice. On the 7th day post-inoculation, the mice received an intravenous injection via the tail vein of a TA-based drug cocktail at a dosage of 5 mg/kg. This cocktail included paclitaxel, docetaxel, doxorubicin, and epirubicin in equal proportions (1:1:1:1, 1.25 mg/kg each drug). On the 28th day, the mice were euthanized using cervical dislocation, and their tumors were collected for analysis. Compared to the sh-NC group, the sh-PFKP group showed a significant reduction in tumor mass (**a**) and tumor volume (**b**) after treatment with the TA-based drugs. Conversely, compared to the OE-NC group, the OE-PFKP group exhibited a significant increase in tumor mass (**a**) and tumor volume (**b**) following the same treatment. **c** and **d** The Kaplan-Meier analysis was conducted to assess the clinical correlation between PFKP mRNA expression levels and relapse-free survival rates in BC cohorts. Using the optimal cutoff value, PFKP expression was categorized into high and low PFKP expression groups. The log-rank test was then applied to calculate the corresponding P-values for survival differences. Notes: Data are presented as mean ± SD. L represents low PFKP expression, H represents high PFKP expression

We modulated PFKP expression in BC cells through gene silencing or overexpression, as verified by WB (Fig. 2a). Silencing PFKP decreased the IC_50_ of TA-based drugs, while overexpression increased the IC_50_ (Fig. 2b). After TA-based drug treatment, apoptosis evaluated by Annexin-V staining (Fig. 2c, Fig. S1a), caspase-3 activity (Fig. S2a, Fig. S1b), and TUNEL assay (Fig. S2b, Fig. S1c) indicated that PFKP promoted resistance of BC cells to TA-based drugs. To investigate long-term effects, MDA-MB-231 and SK-BR-3 cells were subjected to repetitive treatment with paclitaxel, docetaxel, doxorubicin, or epirubicin (5 ng/μL for 24 h, repeated ten cycles). WB analysis revealed that repeated treatments significantly increased PFKP expression compared to WT group (Fig. 2d). In vivo experiments demonstrated that following TA-based drug treatment, tumor weight (Fig. 3a) and tumor volume (Fig. 3b) were reduced in PFKP-silenced groups and increased in PFKP-overexpressing groups compared to controls. Clinically, PFKP^high^ BC patients exhibited lower relapse-free survival rates compared to PFKP^low^ patients following TA-based treatment (Fig. 3c) and other treatments (Fig. 3d). These findings indicate PFKP promotes TA resistance in BC.

### PFKP enhances the formation of CD133^+^ BCSLCs

**Fig. 4:**
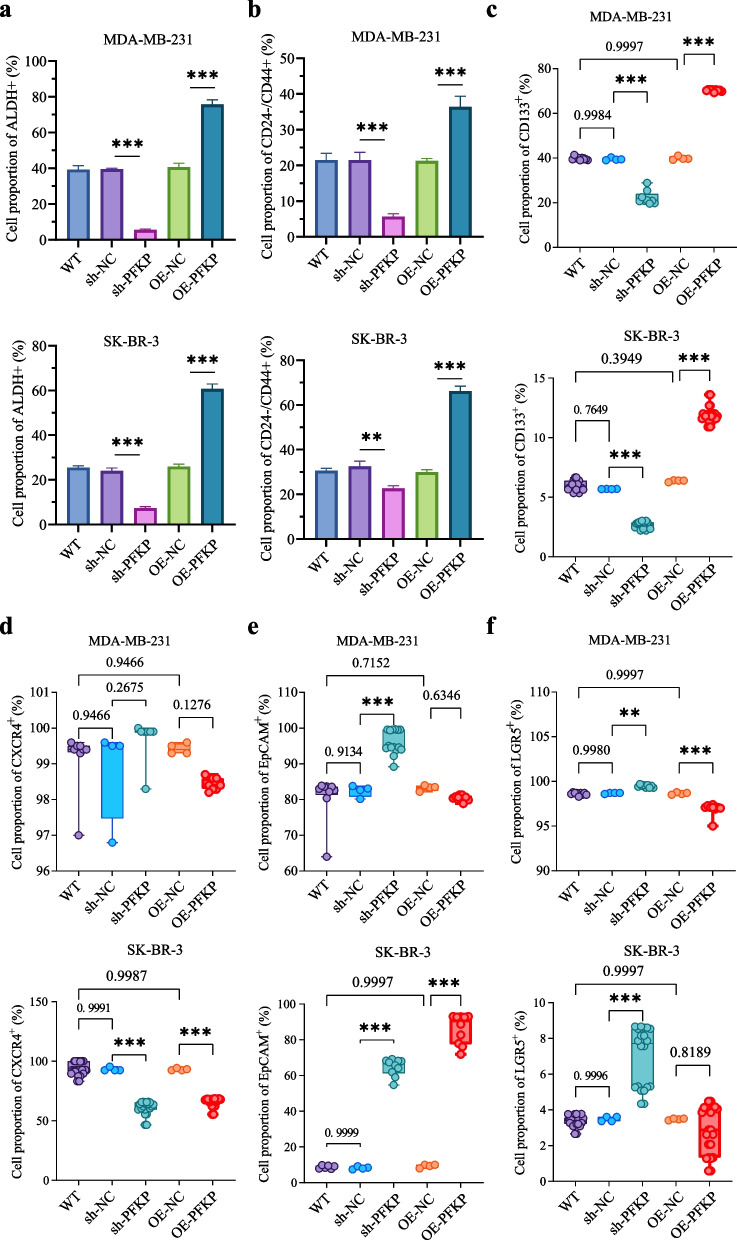
PFKP promotes the formation of CD133^+^ BCSLCs. **a**-**c** In both MDA-MB-231 and SK-BR-3 cells, PFKP knockdown inhibited the formation of ALDH⁺ (**a**), CD24^-^/CD44⁺ (**b**), and CD1133⁺ (**c**) cells, whereas PFKP overexpression promoted the generation of ALDH⁺ (**a**), CD24^-^/CD44⁺ (**b**), and CD1133⁺ (**c**) cell populations. **d** In SK-BR-3 cells, the proportion of CXCR4^+^ cells significantly decreased following either PFKP silencing or overexpression. However, in MDA-MB-231 cells, no significant changes in CXCR4^+^ cells were observed after PFKP silencing or overexpression. **e** In both MDA-MB-231 and SK-BR-3 cells, the proportion of EpCAM^+^ cells significantly increased following PFKP silencing. In contrast, in SK-BR-3 cells, the proportion of EpCAM^+^ cells significantly increased with PFKP overexpression. No significant changes were observed in MDA-MB-231 cells with PFKP overexpression. **f** In both MDA-MB-231 and SK-BR-3 cells, the proportion of LGR5^+^ cells significantly increased following PFKP silencing. In MDA-MB-231 cells, PFKP overexpression significantly decreased the proportion of LGR5^+^ cells. No significant changes were observed in SK-BR-3 cells with PFKP overexpression. Notes: Each experiment was performed at least four times. Data are presented as mean ± SD

To investigate how PFKP contributes to resistance, we extracted chemoresistance-related genes from GeneCards and identified those co-expressed with PFKP. Functional enrichment analysis revealed these genes were enriched in stem cell-related pathways (Fig. S3a). Pearson's correlation analysis showed positive correlation between PFKP mRNA expression and tumor stemness (Fig. S3b), as well as with BCSLC-related markers (Fig. S4). In validation experiments with WT, sh-NC, sh-PFKP, OE-NC, and OE-PFKP BC cells, the proportions of ALDH^+^, CD24^-^/CD44^+^, CD133^+^, EpCAM^+^, CXCR4^+^, and LGR5^+^ cells were measured by flow cytometry (Fig. 4a-f, Fig. S5 and Fig. S6). Notably, silencing PFKP significantly inhibited the formation of CD133^+^ BCSLCs, whereas overexpression enhanced their formation (Fig. 4c).

### CD133^+^ BCSLCs are more resistant to TA-based drugs

**Fig. 5:**
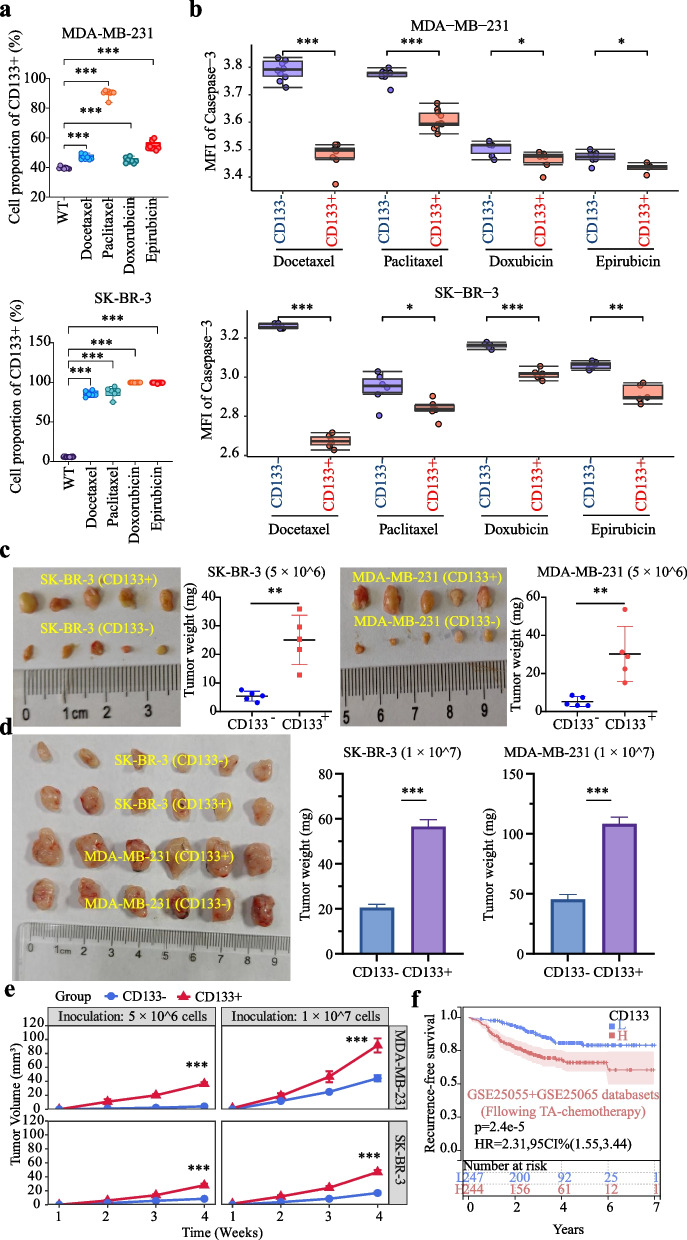
CD133^+^ BCSLCs are resistant to TA-based drugs. **a** In both MDA-MB-231 and SK-BR-3 cells, treatment with TA-based drugs (5 ng/μL of paclitaxel, 5 ng/μL of docetaxel, 2.5 ng/μL of doxorubicin, or 2.5 ng/μL of epirubicin) for 24 h significantly increased the proportion of CD133^+^ cells in viable cells compared to WT controls. **b** In MDA-MB-231 and SK-BR-3 cells, after treatment with TA-based drugs (5 ng/μL of paclitaxel, 5 ng/μL of docetaxel, 2.5 ng/μL of doxorubicin, or 2.5 ng/μL of epirubicin) for 24 h, the mean fluorescence intensity (MFI) of caspase-3 in CD133^+^ cells was significantly lower compared to CD133^-^ cells. **c**-**e** We inoculated CD133^-^ cells (5×10^6^, n=5 or 1×10^7^, n=6) and CD133^+^ cells subcutaneously into the right flank of nude mice. On the 7th day post-inoculation, the mice received an intravenous injection via the tail vein of a TA-based drug cocktail (paclitaxel : docetaxel : doxorubicin : epirubicin = 1 : 1 : 1 : 1, 1.25 mg/kg each drug) at a dosage of 5 mg/kg. On day 28th, the mice were euthanized by cervical dislocation and tumors were excised. Following treatment with the TA-based drugs,, the weight of tumors (**c** and **d**) and tumor volume (**e**) in the CD133^+^ group was significantly greater compared to the CD133^-^ group. **f** High CD133 expression in BC patients is associated with an increased risk of recurrence after TA-based chemotherapy. Notes: Data are presented as mean ± SD. L represents low CD133 expression, H represents high CD133 expression

Compared with untreated WT cells, the proportions of CD133^+^ cells increased in surviving WT cells after TA-based drug treatment for 24 h (Fig. 5a). BC cells were categorized based on CD133 expression using FACS, and each subpopulation was subjected to TA-based drug treatment. Following treatment, the mean fluorescence intensity (MFI) of caspase-3 in CD133^+^ cells was significantly lower than in CD133^-^ cells (Fig. 5b). Furthermore, the CD133^+^ group showed a significant increase in tumor weight (Fig. 5c-d) and tumor volume (Fig. 5e) compared to the CD133^-^ group after TA-based chemotherapy in vivo. Clinically, CD133^high^ BC patients exhibited a notably higher recurrence rate compared to CD133^low^ patients following TA-based chemotherapy (Fig. 5f). These findings suggest CD133^+^ CSLCs are more resistant to TA-based drugs compared to CD133^-^ non-CSLCs.

### PFKP induces the formation of CD133^+^ BCSLCs via activating glycolysis

**Fig. 6:**
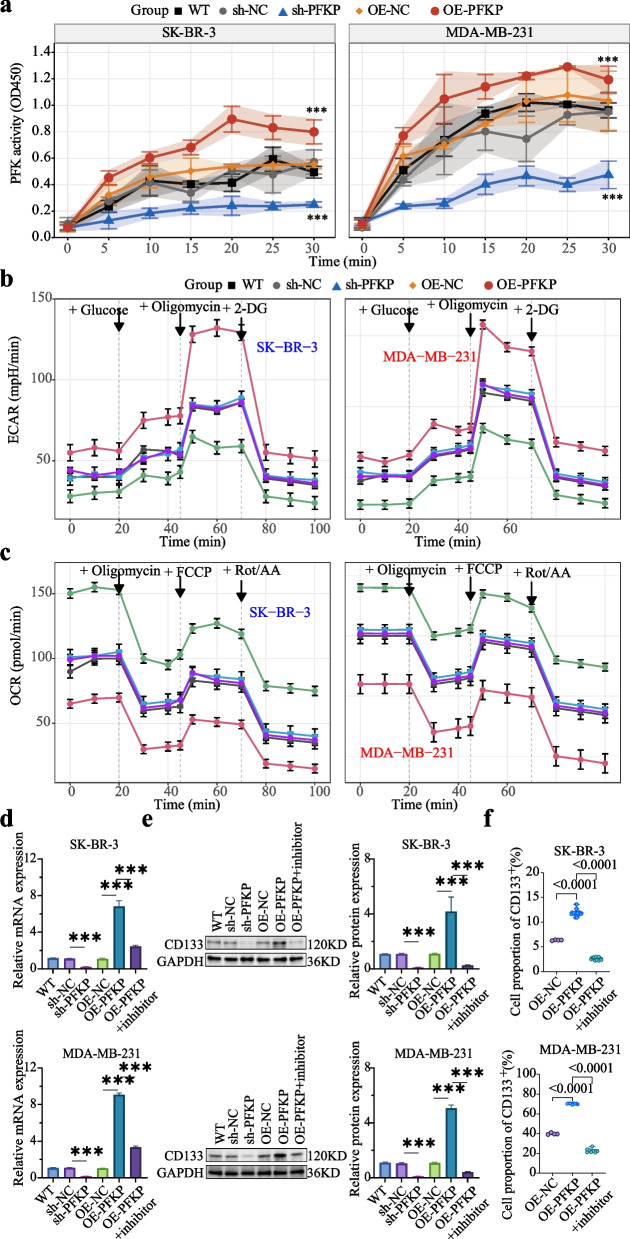
PFKP promotes the formation of CD133^+^ BCSLCs via activating glycolysis. **a** PFKP knockdown inhibited PFK enzyme activity, whereas PFKP overexpression promoted PFK activity. **b** Seahorse analyses demonstrated that PFKP knockdown suppressed extracellular acidification rate (ECAR), while PFKP overexpression elevated ECAR. **c** PFKP knockdown enhanced oxygen consumption rate (OCR), whereas PFKP overexpression reduced OCR activity. **d** and **e** qRT-PCR and WB assays revealed that PFKP knockdown inhibited CD133 expression, while PFKP overexpression promoted CD133 expression. Compared with the PFKP-overexpressing (OE-PFKP) group, treatment with 5 μM glycolysis inhibitor (2-DG) for 48 h significantly repressed CD133 expression in the OE-PFKP +2-DG group. **f** Flow cytometry results suggest that the addition of 5 uM glycolysis inhibitor for 48 h inhibits the promoting effect of PFKP overexpression on the formation of CD133^+^ CSLCs in BC cells. Notes: Each experiment was performed at least three times. Data are presented as mean ± SD

Untargeted metabolomics detected 2,991 metabolites in WT and OE-PFKP SK-BR-3 cells. Compared with WT, 323 metabolites significantly increased and 310 significantly decreased in the OE-PFKP group (Fig. S7a). These differentially expressed metabolites were enriched in glycolysis and Warburg effect pathways (SMPDB database; Fig. S7b). Glucose uptake experiments showed that PFKP overexpression enhances glucose intake, while suppression decreases it (Fig. S7c). Targeted metabolomics showed lower glucose content in OE-PFKP group compared to WT, while sh-PFKP group exhibited an increase. PFKP overexpression increased levels of fructose-6-phosphate, 2-phospho-D-glycerate, phosphoenolpyruvate, and pyruvate, while PFKP silencing reduced these metabolites (Fig. S8a). PFKP overexpression enhanced phosphofructokinase (PFK) activity (Fig. 6a). Seahorse assay demonstrated that PFKP overexpression increased extracellular acidification rate (ECAR) (Fig. 6b) while suppressing oxygen consumption rate (OCR) (Fig. 6c). Targeted metabolomics revealed that PFKP overexpression inhibited oxidative phosphorylation (OXPHOS), while PFKP silencing activated this pathway (Fig. S8b). CCK-8 assay showed that OE-PFKP BC cells became more sensitive to TA-based drugs when glycolysis inhibitors were added, decreasing IC_50_ (Fig. S8c). qRT-PCR, WB (Fig. 6d and e), and flow cytometry (Fig. 6f) demonstrated that glycolysis inhibitors reversed PFKP-induced CD133^+^ BCSLC formation. Together, PFKP contributes to chemoresistance by promoting glycolysis-induced CD133^+^ BCSLC formation.

### CAFs induce the formation of CD133^+^ BCSLCs via CXCL16-ACKR1 axis

**Fig. 7:**
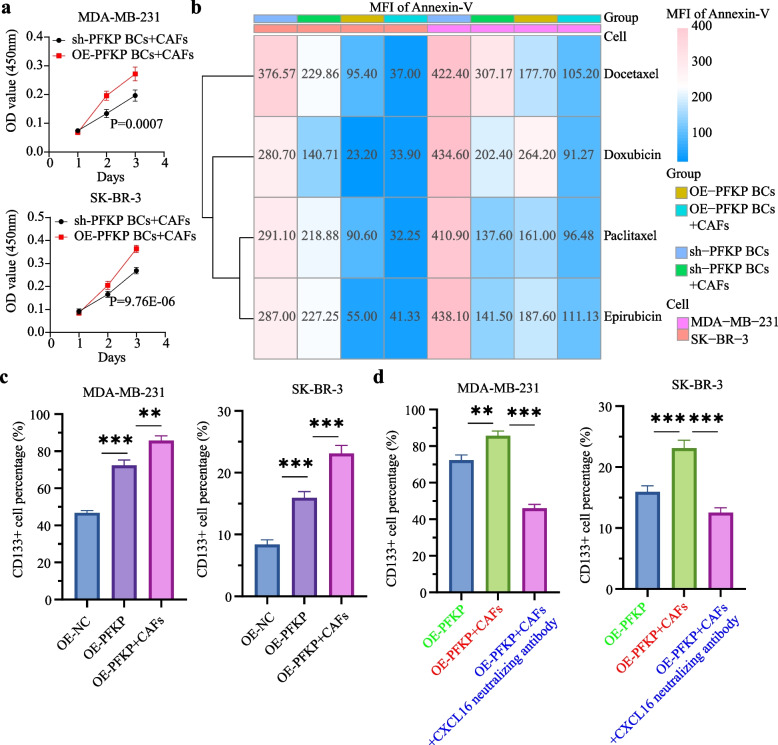
CAFs may promote the formation of CD133^+^ BCSLCs via CXCL16-ACKR1 and HCRT-HCRTR1 axis. **a** CCK-8 assay results indicated that co-cultivation with OE-PFKP BC cells significantly increased the proliferation of CAFs compared to co-cultivation with sh-PFKP BC cells. **b** CCK-8 assay results revealed that the IC_50_ of BC cells was significantly increased in the sh-PFKP BCs + CAFs co-culture group compared to the sh-PFKP BCs group. Similarly, the IC_50_ was significantly increased in the OE-PFKP BCs + CAFs co-culture group compared to the OE-PFKP BCs group. **c** Flow cytometry results showed that the proportion of CD133^+^ cells in BC cells was significantly increased in the OE-PFKP BCs + CAFs co-culture group compared to the OE-PFKP BCs group. **d** Compared with the OE-PFKP BCs + CAFs group, the proportion of CD133^+^ cells was significantly decreased in the OE-PFKP BCs + CAFs + CXCL16 neutralizing antibody group (10 ng/μL, 1 week treatment). Notes: Each experiment was performed at least three times. Data are presented as mean ± SD

In GSE25055 and GSE25065 datasets, we employed 'xCell' to dissect the TME and identify cells associated with CD133 mRNA expression (Fig. S9a). LASSO-Cox analysis identified cells correlated with relapse-free survival (Fig. S9b). Using 'MCPcounter', we further evaluated correlations between these cells and CD133 mRNA expression (Fig. S9c). Correlation analysis revealed a significant positive association between CD133 mRNA and fibroblasts (Fig. S9d). An indirect co-culture system of BC cells with CAFs was established. CCK-8 assay demonstrated that co-cultivation with OE-PFKP BC cells significantly increased the proliferation of CAFs compared to co-cultivation with sh-PFKP BC cells (Fig. 7a). Flow cytometry apoptosis assays indicated that after TA-based drug treatment, compared to non-co-cultured cells, the MFI of Annexin-V and Caspase-3 was markedly lower (Fig. 7b) while the proportion of CD133^+^ BCSLCs was significantly higher (Fig. 7c) in BC cells co-cultured with CAFs. These findings suggest elevated PFKP expression in BC cells enhances CAF proliferation, and these proliferated CAFs enhance chemoresistance by promoting CD133^+^ BCSLC formation. To further elucidate mechanisms, we co-cultured sh-PFKP SK-BR-3 and sh-PFKP MDA-MB-231 cells with CAFs for one week, sorted CD133^-^, CD133^+^, and CAF cells, and performed scRNA-seq. Using "Seurat", subpopulation analysis was performed (Fig. S10a). Using "CellChat", signaling pathways involved in cell communication among CAF, CD133^-^, and CD133^+^ subpopulations were analyzed (Fig. S10b), which included CXCL signaling (CXCL10-ACKR1 and CXCL16-ACKR1 axes) (Fig. S10c). Flow cytometry showed that the proportion of CD133^+^ BCSLCs was significantly decreased in the OE-PFKP + CAFs + CXCL16 neutralizing antibody group compared with the OE-PFKP + CAFs group (Fig. 7d). These findings indicate that CAFs communicate with BC cells via the CXCL16-ACKR1 axis, promoting CD133^+^ BCSLC formation.

## Discussion

PFKP has been recognized as an oncogene with elevated expression in multiple cancers [20]. We observed elevated PFKP expression in BC, especially in basal-type BC. This upregulation enhances intratumoral CD133^+^ cells in a glycolysis-dependent manner. Moreover, BC cells with high PFKP expression promote CAF proliferation, and CAF-mediated activation of the CXCL16/CXCR6 signaling axis contributes to CD133^+^ BCSC generation.

The efficacy of chemotherapy is often compromised by resistance mechanisms that are either intrinsic at diagnosis or acquired during treatment [21]. We identified PFKP as a key contributor to TA resistance in BC. Notably, TA-based chemotherapeutic agents induced PFKP expression in BC cells, suggesting a feedback mechanism that may further impair treatment response. These findings highlight a dual role for PFKP: supporting pre-existing resistance mechanisms and being upregulated by treatment, potentially amplifying resistance over time.

CSLCs represent a key feature of tumor chemoresistance [22]. While suppressing PFKP reduces CSLC formation in liver cancer [23] and glioma [24], its specific role in BC was unclear. We found that PFKP promotes formation of CD133^+^ CSLCs in BC. Importantly, CD133^+^ BCSLCs are more resistant to TA-based drugs than CD133^-^ non-BCSLCs. Studies indicate CD133^+^ CSLC formation in glioblastoma primarily relies on OXPHOS [25], while in liver cancer it depends on glycolysis [26]. Our findings demonstrate PFKP triggers metabolic reprogramming from OXPHOS to glycolysis in BC, facilitating CD133^+^ BCSLC formation. In hepatocellular carcinoma, CD133^+^ cells exhibit enhanced post-chemotherapy survival through AKT/PKB and Bcl-2 pathway activation [27]. In gastric cancer, the the interaction between CD133 and PI3K-p85 activates PI3K/AKT signaling, reducing chemosensitivity [28]. These findings suggest that PFKP-mediated promotion of CD133^+^ CSLCs contributes to chemoresistance.

Tumor cells facilitate conversion of normal fibroblasts into CAFs and stimulate their proliferation [29]. Our findings show that BC cells with elevated PFKP significantly increase CAF proliferation. CAFs contribute to chemoresistance and enhance CD133^+^ CSLC formation in BC cells through the CXCL16/CXCR6 axis. CXCL16 binding to CXCR6 triggers PI3K/AKT, ERK, and MAPK signaling, activating mTOR and NF-κB [30]. mTOR pathway activation leads to CD133^+^ CSLC formation in nasopharyngeal [31], colorectal [32], and pancreatic cancer [33], while NF-κB pathway blockade inhibits this formation in hepatocellular carcinoma [34, 35] and enhances chemotherapy sensitivity [36, 37]. These findings suggest that CAFs promote the formation of CD133^+^ BCSLCs via the CXCL16/CXCR6 axis, thereby contributing to chemoresistance.

### Limitations

The molecular mechanism underlying accelerated CAF proliferation when co-cultured with PFKP-overexpressing BC cells remains undetermined. Future investigations will focus on functional assays using conditioned medium combined with cytokine array screening and metabolomic profiling to identify key paracrine factors.

The dosing strategy of the TA-based four-drug cocktail (1:1:1:1 ratio) requires contextualization. The reported dose of 5 mg/kg represents total mass, with each drug at 1.25 mg/kg. Whether this low-dose combination sufficiently mimics clinical chemotherapy pressure remains unresolved. Future studies should validate efficacy under dose regimens that more closely mirror clinical practice.

The incomplete characterization of PFKP-mediated regulation of distinct CSC subsets is another limitation. While PFKP enhances glycolysis to promote CD133^+^ BCSCs expansion, inconsistent alterations in other stemness markers (CD44^+^, EpCAM^+^, LGR5^+^) upon PFKP manipulation have not been fully elucidated. This may stem from context-dependent metabolic preferences of different CSC subsets.

### Conclusions

CD133^+^ BCSLCs contribute to TA resistance in BC. Intracellularly, PFKP promotes CD133^+^ BCSLC formation by activating glycolysis. Extracellularly, PFKP^high^ BC cells promote CAF proliferation, and these CAFs facilitate CD133^+^ BCSLC formation via the CXCL16/CXCR6 axis. Targeting PFKP or CD133 may offer a promising therapeutic approach for BC, especially in patients with elevated PFKP.

## Materials and methods

Materials and methods are available in the supplementary materials.

## Supplementary Information

Supplementary Material 1.

## Data Availability

The datasets supporting this manuscript’ conclusions are available in the ArrayExpress database (https://www.ebi.ac.uk/biostudies/arrayexpress, No. E-MTAB-14538), The Cancer Genome Atlas Program (TCGA, https://portal.gdc.cancer.gov/, data access and download were performed from June to October 2022), Gene Expression Omnibus (GEO, https://www.ncbi.nlm.nih.gov/geo/, data access and download were performed from June to October 2022) and cBio Cancer Genomics Portal (cBioPortal, https://www.cbioportal.org/, data access and download were performed from June to October 2022) databases.
