# Supplementary material for: PFKP is required for chemoresistant phenotype of breast cancer through modulating the formation of CD133+ cancer stem like cells
Source: Mol Biomed. 2026 Apr 23;7:56. doi: 10.1186/s43556-026-00454-z (PMC13106758; doi:10.1186/s43556-026-00454-z)
Supplement: Supplementary file 1 — Supplementary Material 1. [file 43556_2026_454_MOESM1_ESM.pdf]

Materials and methods

Public omics datasets for differential expression analysis

Tumor purity was calculated using the R package “ESTIMATE” with tumor purity = cos (0.6049872018 + 0.0001467884 × (ESTIMATE Score = Stromal Score + Immune Score))(1). Samples with a tumor purity of 30% or higher were included in this study, consistent with previous reports(2, 3). In this study, the enrolled sequencing profiles of mRNA and protein were retrieved from The Cancer Genome Atlas Program (TCGA, https://portal.gdc.cancer.gov/), Gene Expression Omnibus (GEO, https://www.ncbi.nlm.nih.gov/geo/) and cBio Cancer Genomics Portal (cBioPortal, https://www.cbioportal.org/) databases (Table. S1). The R packages "limma-voom" and "limma", along with the "Wilcoxon rank-sum test", were used to screen differential expressed genes (DEGs) (Table. S2).

In-house BC cohort

From January 2020 to August 2022, basal-type tumors (N=25), non-basal-type tumors (N=40), and adjacent normal tissues (N=25) were obtained from surgeries at the Affiliated Hospital of Jiangnan University (Wuxi, China) (Table. S4). All patients involved in this study were newly diagnosed and had not undergone any prior treatment. Written informed consent was obtained from all participants. This study was conducted with the approval of the Institutional Review Board and in compliance with the Declaration of Helsinki. The Ethics Committee of the Affiliated Hospital of Jiangnan University approved this study (License number: LS2020064).

Cell lines and chemicals

The human MDA-MB-231 and SK-BR-3 cell lines were purchased from Zhongqiao Xinzhou Biotechnology Co., LTD (Shanghai, China), and were cultured in DMEM medium supplemented with 10% FBS and 1% penicillin-streptomycin. The human BC-associated fibroblasts (ZQY042) were purchased from Zhongqiao Xinzhou Biotechnology Co., Ltd, and were cultured in corresponding complete culture medium (PCM-H-216). All cell cultures were maintained in a humidified incubator set at 37°C with 5% CO2. Paclitaxel (HY-B0015), docetaxel (HY-B0011),

doxorubicin (HY-15142), epirubicin (HY-13624A), and 2-Deoxy-D-glucose (HY-13966, Glycolysis inhibitors) were all procured from MedChemExpress (Shanghai, China).

Cell transfection

The PFKP-coding sequences were inserted into the pLVX-mCMV-tdTomato-puromycin (puro) vector (BR560, Hunan Fenghui Biotechnology Co., Ltd, China), creating a lentiviral plasmid designed for PFKP overexpression (OE-PFKP). Additionally, short hairpin RNA (shRNA) oligonucleotides targeting the human PFKP gene were incorporated into the pLKO.1-mCherry-Puro vector (ZT413, Hunan Fenghui Biotechnology Co., Ltd, China), yielding a lentiviral plasmid for PFKP knockdown (sh-PFKP). These plasmids and corresponding negative control (NC) plasmids were purchased from Fenghui Biotechnology Co., LTD (Changsha, Hunan Province, China). The pLTR-G lentiviral VSVG envelope protein expression plasmid (FH1657, Fenghui Biotechnology Co., Ltd) and the lentiviral packaging vector pSPAX2 (Fenghui Biotechnology Co., Ltd) were utilized for the construction of lentiviruses. Puro was employed to select cell lines infected by the lentivirus.

CCK-8 assay

Cells were seeded in 96-well plates at a density of 1000 cells per well and incubated for 24 hours. Following this, 90 µl of serum-free medium was mixed with 10 µl of CCK-8 reagent (PC001, proteinbio, Nanjing, China), and applied to each well for 2 hours according to the manufacturer's instructions. Absorbance readings were taken at 450 nm at 24-hour intervals to monitor cell viability.

Transwell assay

Cells were plated in transwell chambers with 8 µm pore membranes. For invasion assays, the upper chamber's membrane was precoated with 60 µl Matrigel and incubated at 37°C with 5% CO2 for 2 hours. Then, 1×10^6 cells in 300 µl serum-free medium were added to the upper chamber, while 800 µl of medium containing 20% FBS was placed in the lower chamber. For migration assays, 5×10^5 cells in 300 µl serum-free medium were seeded in the upper chamber without

Matrigel, with 800 μl of 20% FBS medium in the lower chamber. Both assays involved a 12–24 hour incubation at 37°C in 5% CO2. After incubation, we fixed the cells with 100% methanol for 30 minutes at room temperature. This fixation step is crucial as it stabilizes the cellular structures, preventing degradation and ensuring the cells remain in place for accurate staining and counting. Following fixation, we stained the cells with crystal violet for 1 hour. The crystal violet dye penetrates the cells, staining their nuclei and cytoplasm, making them clearly visible under a light microscope. This staining process facilitates the quantification of migrated or invaded cells by allowing clear visualization. Lastly, the upper membrane cells were scraped off, and images from three random fields were taken under a microscope and analyzed using Photoshop software.

Median inhibitory concentration (IC_{50})

Cells were seeded in 96-well plates at a density of 5×10^3 cells per well and incubated for 24 hours. Following this, BC cells were treated with TA-based drugs at 0-32ng/ul for 24 hours. Then 90 μl of serum-free medium was mixed with 10 μl of CCK-8 reagent and applied to each well for 2 hours. Absorbance readings were taken at 450 nm.

Real-time fluorescence quantitative PCR (RT-qPCR)

Total RNA was extracted using trizol and was reverse-transcribed using HiScript III 1st Strand cDNA Synthesis Kit (+gDNA wiper) (R312-01, Vazyme, Nanjing, China). ChamQ SYBR qPCR Master Mix (High ROX Premixed, Vazyme) was used for PCR. The primers used are listed in Table. S4.

Western blot (WB)

Tissue and cells were lysed using RIPA buffer supplemented with PMSF (RIPA : PMSF = 100 : 1). Equal protein amounts were resolved by SDS-PAGE and transferred to PVDF membranes. Signals were captured using Enhanced ECL Prime (PECL08, proteinbio) with the Amersham Imager 600 and subsequently quantified utilizing ImageJ software. GAPDH served as the internal control for normalization. HRP-IgG was used as secondary antibody. The antibodies used are listed in Table. S5.

Apoptosis detection

After treating 5 × 10^6 cells with TA-based drugs (including 5 μg/ml paclitaxel, 5 μg/ml docetaxel, 2.5 μg/ml doxorubicin, or 2.5 μg/ml epirubicin, respectively) or without TA-based drugs for 24 hours, apoptosis of the cells was assessed using Annexin V-APC (640920, BioLegend), SuperView™ 488 Caspase-3 (PS6007L, ProteinBio), and/or the YF®488 TUNEL Assay Apoptosis Detection Kit (PT0488-50T, ProteinBio). The APC channel (excitation wavelength of 633nm, emission wavelength of 660nm) was utilized for detecting Annexin-V, and the FITC channel (excitation wavelength of 488nm, emission wavelength of 525nm) was used for detecting caspase-3 or TUNEL via a flow cytometer (BD FACSLCelesta™).

Fluorescence-activated cell sorting (FACS)

The APC channel is used to detect CXCR4, EpCAM, and LGR5, while the FITC channel is used to detect CD24, CD44, and CD133. In OE-PFKP BC cells, cells were categorized into distinct subpopulations, $CD24^-$ BCSLCs, $CD24^+$ non-BCSLCs, $CD44^-$ non-BCSLCs, $CD44^+$ BCSLCs, $CD133^-$ non-BCSLCs, $CD133^+$ BCSLCs, $EpCAM^-$ non-BCSLCs, $EpCAM^+$ BCSLCs, $CXCR4^-$ non-BCSLCs, $CXCR4^+$ BCSLCs, $LGR5^-$ non-BCSLCs, and $LGR5^+$ BCSLCs via using a flow cytometry via FACS. The PE channel is used to sort sh-PFKP or OE-PFKP BC cells using a flow cytometry via FACS.

Cell co-culture system

CAFs were indirectly co-cultured with or without sh-PFKP or OE-PFKP BC cells for one week using a 0.4um transwell chamber, after which the CAFs were harvested, and their proliferation was assessed using the CCK-8 assay. BC cells were directly co-cultured with CAFs in the presence or absence of a CXCL16 neutralizing antibody (10 ng/μL, Cat. No. MA5-23952, Thermo Fisher Scientific) for one week. Subsequently, the BC cells were isolated and sorted via flow cytometry. The sorted BC cells were then used to detect markers of BCSLCs with a flow cytometer. Additionally, the sorted BC cells were treated with TA-based drugs for 24 hours, after which Annexin-V and caspase-3 were measured using a flow cytometer. Moreover, the sorted BC cells were used for single-cell RNA sequencing (scRNA-seq).

Seahorse assay

Cellular extracellular acidification rate (ECAR) and oxygen consumption rate (OCR) were measured on a Seahorse XFe24 Flux Analyzer (Seahorse Bioscience, Agilent) with the corresponding glycolytic (Cat. #103020–100) and mitochondrial (Cat. #103015–100) stress test kits. In brief, 1×10^4 cells were plated in 96-well XF plates as directed, cultured at 37 °C in pH 7.4 XF base medium, and treated sequentially with glucose (10 mM), glutamine (1 mM), 2-DG (50 mM), and oligomycin (1 μM) at specified time points. Data acquisition and plotting were performed using Seahorse XF24 software.

Phosphofructokinase (PFK) activity assay

Phosphofructokinase (PFK) Activity Assay: The enzymatic activity of PFK was measured using a commercial colorimetric assay kit (MAK093, Sigma-Aldrich, St. Louis, MO, USA) following the manufacturer's instructions; this assay quantifies PFK activity by coupling fructose-1,6-bisphosphate (F-1,6-BP) production to NADH oxidation, with the reaction monitored via the decrease in absorbance at 450 nm. Briefly, assay buffer, developer, enzyme mix, and substrate mix were prepared as per the kit protocol, and cell lysates or tissue homogenates were used as enzyme sources, with their protein concentrations determined using a BCA protein assay kit (Pierce, Thermo Fisher Scientific, Waltham, MA, USA) for activity normalization; a master reaction mix containing assay buffer, developer, enzyme mix, and NADH was prepared on ice, followed by mixing 50 μL of this master mix with 20 μL of sample or assay buffer (blank) in a clear 96-well microplate, initiating the reaction by adding 30 μL of substrate mix (containing fructose-6-phosphate and ATP), immediately mixing the plate, and recording the absorbance at 450 nm every minute for 30-40 minutes at 37°C using a microplate reader (Molecular Devices, SpectraMax i3x) with all assays performed in triplicate. PFK activity was calculated from the linear portion of the kinetic curve, where one unit of PFK is defined as the amount of enzyme generating 1.0 μmole of F-1,6-BP per minute at 37°C under the assay conditions, and the activity was expressed as milliunits per milligram of total protein (mU/mg protein) using the formula: Activity = (ΔA_{450}/min × Reaction Volume × Dilution Factor) / (ε × Pathlength × Sample Volume × Protein Concentration) (ΔA_{450}/min: average change in

absorbance per minute; ε: extinction coefficient of NADH, 6.22×10^3 $M^{-1}cm^{-1}$; pathlength corrected for microplate well volume).

Evaluation of cancer stemness

The gene expression-based stemness index (mRNAsi) is a machine learning algorithm based on one-class logistic regression (OCLR) that uses a trained stemness index model to evaluate the cancer stemness of tissue samples(4). The mRNAsi was calculated for each sample within the respective datasets in the TCGA and METABRIC datasets. Pearson's r correlations between PFKP mRNA expression and mRNAsi were calculated.

Screening for chemo-resistance related genes and PFKP co-expressed genes

Using the keyword 'chemoresistance', 5,056 genes were identified in the GeneCards database (https://www.genecards.org/) with a selection criterion of a relevance score ≥ 0.1. In the TCGA, METABRIC, GSE31448, GSE65194, GSE87049, and GSE78958 datasets, genes co-expressed with PFKP mRNA were calculated using Pearson's r method. The filtering criteria were set to $r \geq 0.2$ and $P \leq 0.05$. Subsequently, functional enrichment analysis was conducted based on the C2: curated gene sets.

Detection of BCSLCs

AldeRed® ALDH Detection Kit (Cat. No. SCR150, Sigma-Aldrich), FITC anti-human CD24 antibody (W20001B, BioLegend), FITC anti-human CD44 antibody (C44Mab-5, BioLegend), FITC anti-human CD133 antibody (W6B3C1, BioLegend), APC anti-human EpCAM antibody (9C4, BioLegend), APC anti-human CD184 (CXCR4) antibody (12G5, BioLegend) and APC anti-human LGR5 (GPR49) antibody (SA222C5, BioLegend) were used to detect the proportions of different phenotypic BCSLCs in MDA-MB-231 and SK-BR-3 cells via using the PE, APC and FITC channel of a flow cytometer.

Glucose uptake detection

2-NBDG (HY-116215, MedChempress) is a standard fluorescently labeled glucose for studying glucose uptake in cells. We treated 1×10^6 cells with 5 nM 2-NBDG for 2 hours and then detected the uptake of 2-NBDG using the FITC channel of a flow cytometer.

Calculation of CAF proportion in tumor tissue

The R software package IOBR integrates eight published methods for decoding the TME context, including xCell, MCPcounter, CIBERSORT, TIMER, ESTIMATE, EPIC, IPS, and quanTIseq(5). In the GSE25055 and GSE25065 datasets, xCell and MCPcounter were utilized to calculate the proportion of CAFs within tumors.

Metabolomics detection in our in-house samples

Ten basal-type tumors, 28 non-basal-type tumors, and 12 adjacent normal tissues were used for targeted metabolomics. Non-targeted metabolomics was performed in WT and OE-PFKP SK-BR-3 cells. Targeted metabolomics was also performed in sh-PFKP, OE-PFKP, and WT cells. Metabolomics was performed by Maiwei Metabolic Biotechnology Co., LTD (Wuhan, China) using a Liquid Chromatography-Tandem Mass Spectrometry (LC-MS/MS) platform and a proprietary database. The data acquisition was performed using an Ultra Performance Liquid Chromatography (UPLC) system (Waters ACQUITY H-ClassD, FUJIFILM Diosynth Biotechnologies, USA) coupled with Tandem Mass Spectrometry (QTRAP® 6500^+, SCIEX, Shanghai, China). MetaboAnalyst5.0 was used to analyze the proteomics data in this study(6). First, the samples and test items with >50% missing values were deleted. Then, the missing values were completed using the "KNN" function in the R package "impute". A pooled sample of normalized data from the standard substance group, with square root transformation and auto-scaling applied. Fold change (FC) was calculated as the ratio between the two group means for differential expressed metabolites analysis with P value < 0.05 via Student's t-test. The R package UMAP was utilized for dimensionality reduction and visualization.

scRNA-seq

CAFs were directly co-cultured with sh-PFKP SK-BR-3 or MDA-MB-231 cells for one week, after which the CAFs and BC cells were harvested via FACS in PE channel. FITC anti-human CD133 antibody was used to sort $CD133^-$ and $CD133^+$ cells in BC via FACS. CAFs, $CD133^-$ (SK-BR-3 and MDA-MB-231 mixed) and $CD133^+$ (SK-BR-3 and MDA-MB-231 mixed) cells were used for scRNA-seq. scRNA-seq libraries were prepared using the Single Cell 3′ Reagent Kit v3.1 from

10x Genomics according to the manufacturer's protocol. The process started with loading cells, gel beads, and partitioning oil onto a Chromium Next GEM Chip, aiming to recover approximately 10,000 cells per sample. The chip was processed in a Chromium Controller to create Gel Beads-in-Emulsion (GEMs). Reverse transcription was performed using the GEM-RT protocol on a PCR cycler. Following this, the GEMs were disrupted with Recovery Agent, and the cDNA was purified using DynaBeads MyOne Silane beads. The cDNA was then amplified via PCR for 11 cycles and further purified with SPRIselect magnetic beads. For library preparation, 25% of the cDNA yield was used. Library quality was assessed on the Agilent TapeStation 4200, and yield was quantified by qPCR with the KAPA Library Quantification Kit. Sequencing was outsourced to Novogene, targeting 20,000 paired reads per cell on an Illumina NovaSeq 2000 platform. Data were deposited in the ArrayExpress database (https://www.ebi.ac.uk/biostudies/arrayexpress, No. E-MTAB-14538, date of public release: 2025-09-30).

Cell-cell communication analysis

We utilized the R package 'CellChat'(7) to explore the cell-cell communication networks. By identifying the overexpressed ligands and receptors within cellular groups, we inferred the intercellular communication networks and conducted network analysis using the visualization tools provided by 'CellChat'.

Animal experiment

BALB/c nude mice (4-6 weeks old, weighing 25 to 30 g) were purchased from Shanghai Jihui Laboratory Animal Co., Ltd., which holds a valid laboratory animal production license with the number SCXK (Shanghai) 2022-0009. All animal experiments were conducted with the approval of the Ethics Committee of the Affiliated Hospital of Jiangnan University (No.20220009025212). A total of 1-2 × 10^7 sh-NC, sh-PFKP, OE-NC, and OE-PFKP cells were inoculated subcutaneously into the right flank of each mouse. Additionally, this study employed FACS to sort $CD133^-$ and $CD133^+$ cells from MDA-MB-231 and SK-BR-3 cells. A total of 5-10 × 10^6 $CD133^-$ or $CD133^+$ cells were then inoculated subcutaneously into the right flank of each mouse. On day 7th, the mice received a tail vein injection of a TA-based drug

cocktail (5 mg/kg), consisting of paclitaxel, docetaxel, doxorubicin, and epirubicin at a 1:1:1:1 ratio. On day 28th, the mice were euthanized by cervical dislocation, and the tumors were harvested.

Statistical analysis

Statistical software GraphPad Prism 8 and FlowJo v10.8.1_CL were used for statistical analysis. Results are presented as the mean±standard deviation (SD). For pairwise comparisons between two independent groups, the non-parametric Mann–Whitney U test was performed. For comparisons involving three or more independent groups, one-way analysis of variance (ANOVA) was first conducted; where a significant main effect was observed, a post-hoc test was subsequently applied to identify specific group differences. A $P \leq 0.05$ was considered statistically significant.

Reference

1. Yoshihara K, Shahmoradgoli M, Martinez E, Vegesna R, Kim H, Torres-Garcia W, et al. Inferring tumour purity and stromal and immune cell admixture from expression data. Nat Commun. 2013;4:2612. https://doi.org/10.1038/ncomms3612.
2. Jiang YZ, Ma D, Suo C, Shi J, Xue M, Hu X, et al. Genomic and Transcriptomic Landscape of Triple-Negative Breast Cancers: Subtypes and Treatment Strategies. Cancer Cell. 2019;35(3):428-40 e5. https://doi.org/10.1016/j.ccell.2019.02.001.
3. Gong Y, Ji P, Yang YS, Xie S, Yu TJ, Xiao Y, et al. Metabolic-Pathway-Based Subtyping of Triple-Negative Breast Cancer Reveals Potential Therapeutic Targets. Cell Metab. 2021;33(1):51-64 e9. https://doi.org/10.1016/j.cmet.2020.10.012.
4. Malta TM, Sokolov A, Gentles AJ, Burzykowski T, Poisson L, Weinstein JN, et al. Machine Learning Identifies Stemness Features Associated with Oncogenic Dedifferentiation. Cell. 2018;173(2):338-54 e15. https://doi.org/10.1016/j.cell.2018.03.034.
5. Zeng D, Ye Z, Shen R, Yu G, Wu J, Xiong Y, et al. IOBR: Multi-Omics Immuno-Oncology Biological Research to Decode Tumor Microenvironment and Signatures. Front Immunol. 2021;12:687975. https://doi.org/10.3389/fimmu.2021.687975.
6. Pang Z, Chong J, Zhou G, de Lima Morais DA, Chang L, Barrette M, et al. MetaboAnalyst 5.0: narrowing the gap between raw spectra and functional insights. Nucleic Acids Res. 2021;49(W1):W388-W96. https://doi.org/10.1093/nar/gkab382.

7. Jin S, Guerrero-Juarez CF, Zhang L, Chang I, Ramos R, Kuan CH, et al. Inference and analysis of cell-cell communication using CellChat. Nat Commun. 2021;12(1):1088. https://doi.org/10.1038/s41467-021-21246-9.

Supplementary Figure legends

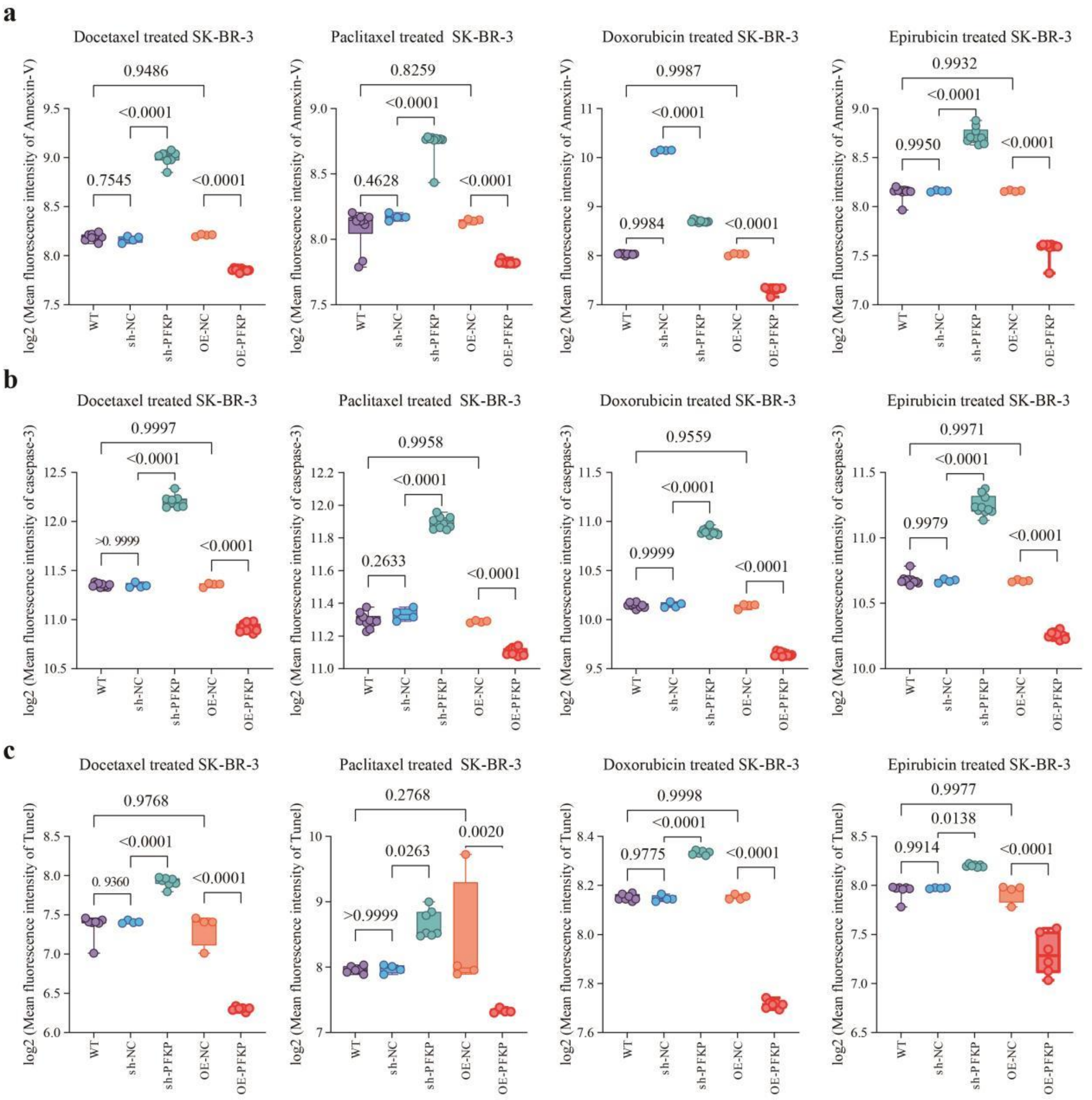

Fig. S1. PFKP promotes resistance of BC cells to TA-based drugs in SK-BR-3 cells.

(a-c) After treating 5 × 10^6 WT, sh-NC, sh-PFKP, OE-NC, and OE-PFKP SK-BR-3 cells with TA-based drugs (including 5 µg/ml paclitaxel, 5 µg/ml docetaxel, 2.5 µg/ml doxorubicin, or 2.5 µg/ml epirubicin) or without these drugs for 24 hours, apoptosis was assessed using three different methods: (a) Annexin-V staining, (b) caspase-3 staining, and (c) TUNEL staining, all performed via flow cytometry. Notes: Each experiment was conducted at least four times. Data are presented as mean ± SD.

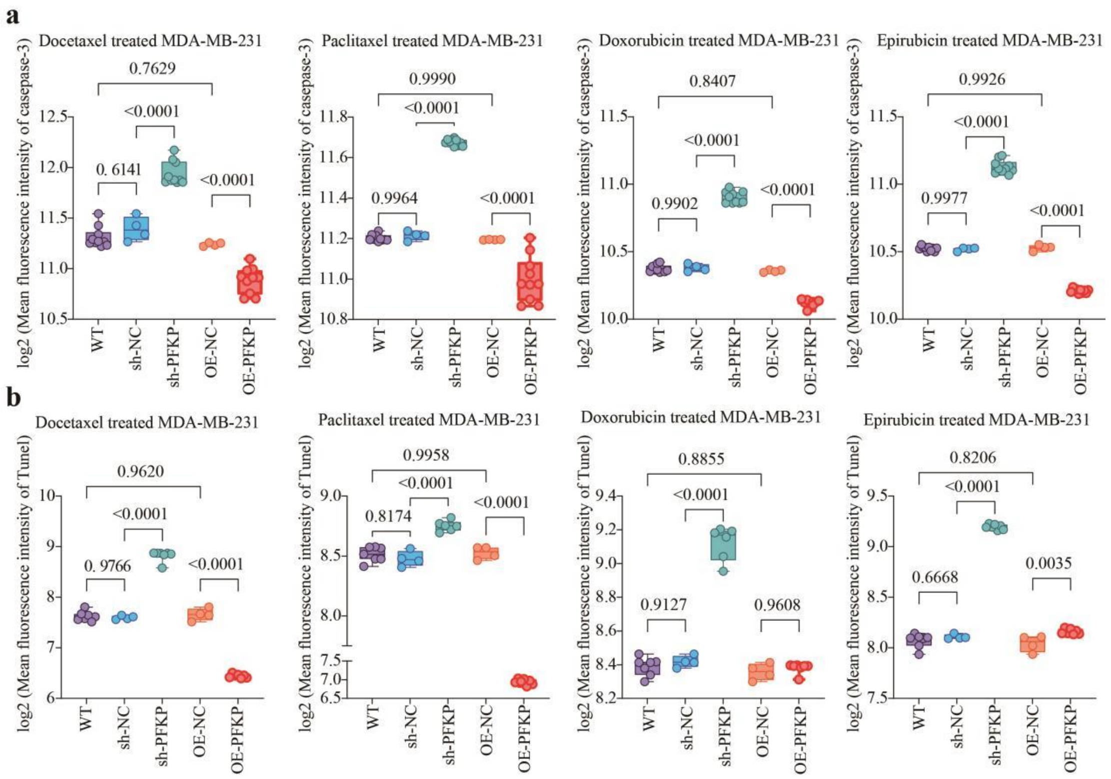

Fig. S2. Effects of TA-based drugs on apoptosis in MDA-MB-231 cells.

(a and b) After treating 5 × 10^6 cells with TA-based drugs (including 5 μg/ml paclitaxel, 5 μg/ml docetaxel, 2.5 μg/ml doxorubicin, or 2.5 μg/ml epirubicin) or without TA-based drugs for 24 hours, apoptosis was assessed using caspase-3 staining (a) and TUNEL staining (b) via flow cytometry in MDA-MB-231 cells. Each experiment was performed at least four times.

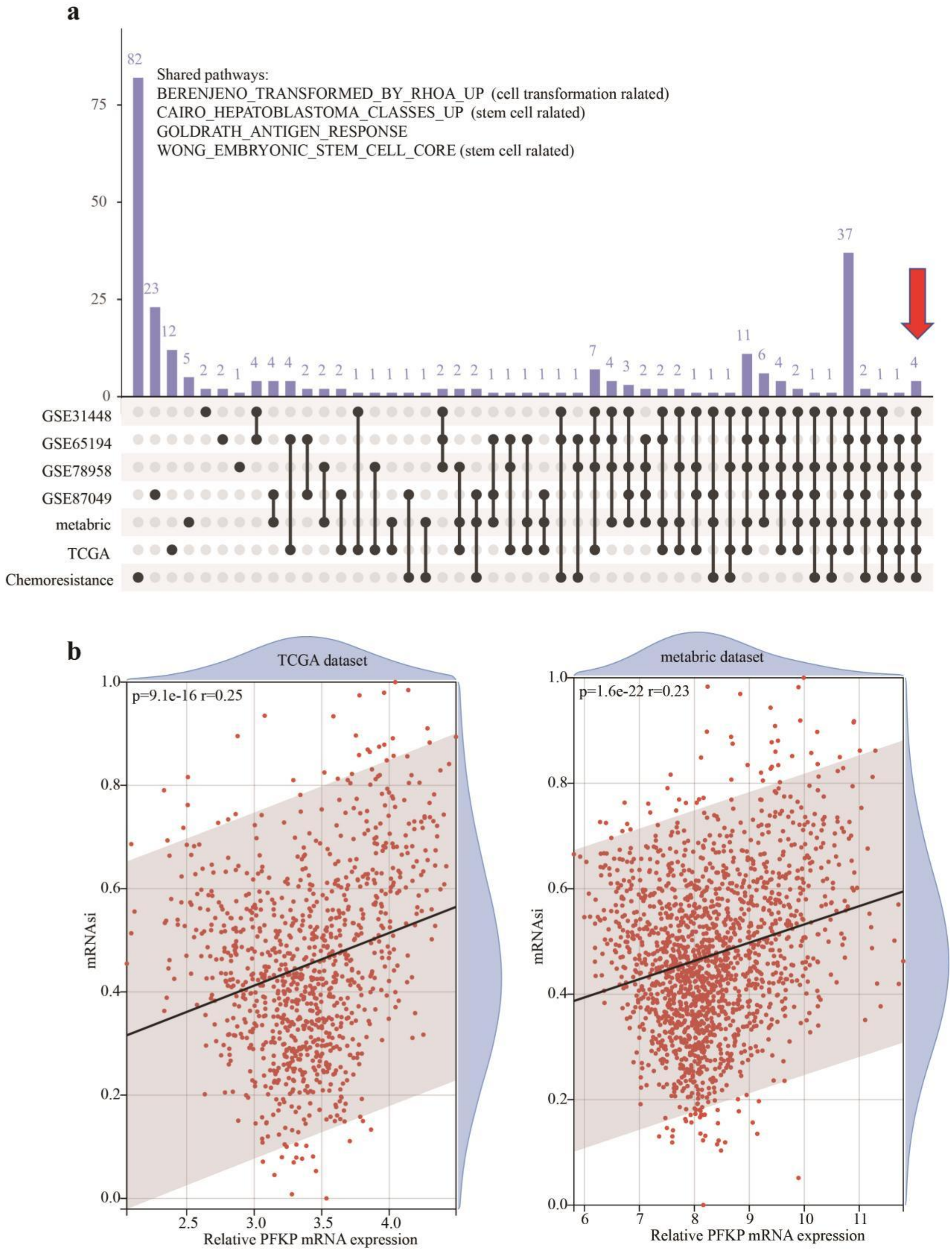

Fig. S3. The molecular pathways that PFKP may modulate.

(a) Functional enrichment analysis results indicated that genes associated with chemoresistance and genes co-expressed with PFKP were commonly enriched in stem cell-related pathways. (b) Pearson's correlation analysis showing a positive correlation between PFKP mRNA expression and tumor stemness in BC tumors from the TCGA and METABRIC cohorts.

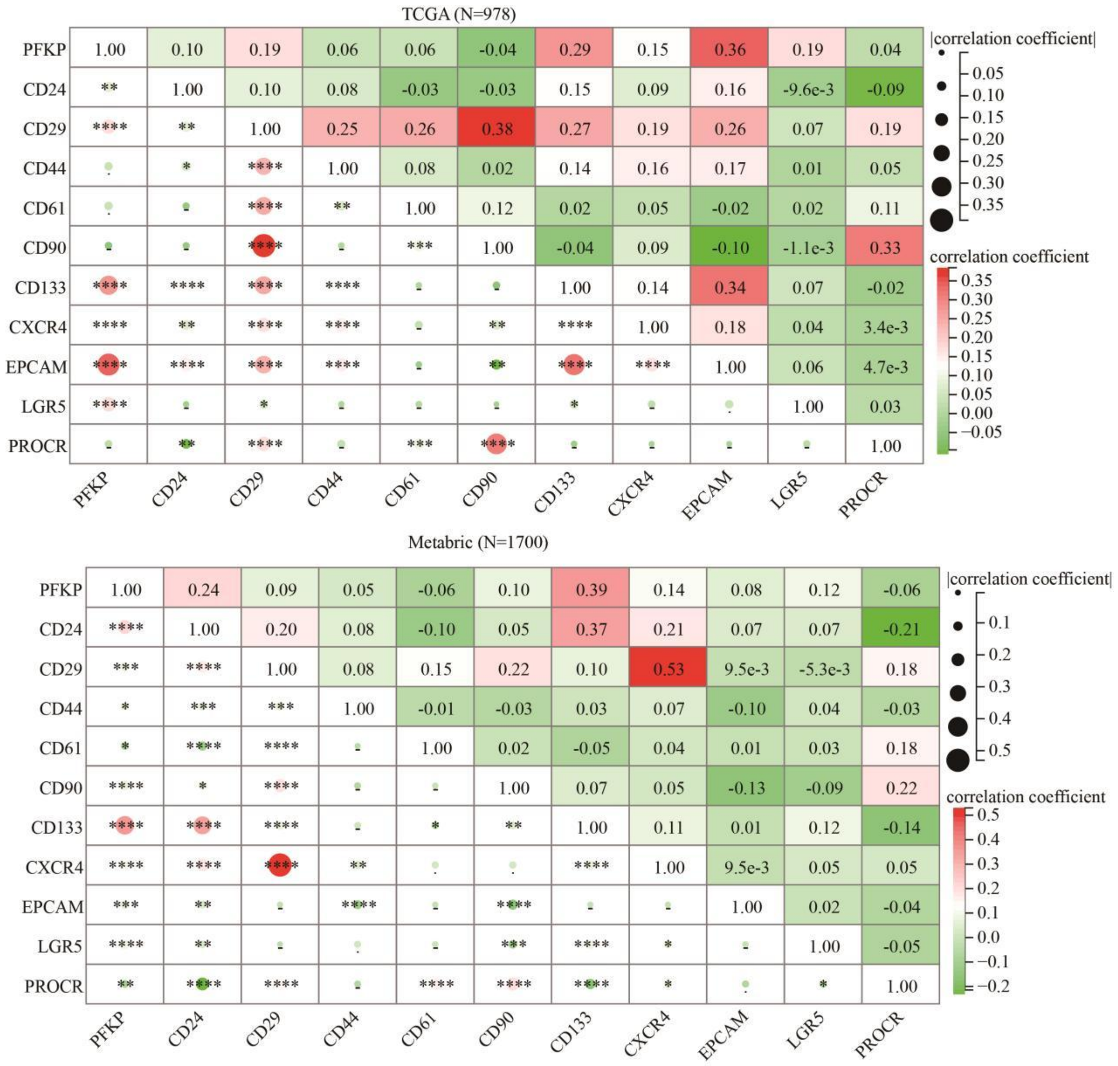

Fig. S4. Correlation between PFKP expression and CSLC-related markers in multiple breast cancer datasets.

Positive correlation between PFKP mRNA expression and cancer stem-like cell (CSLC)-related markers within the METABRIC and TCGA datasets.

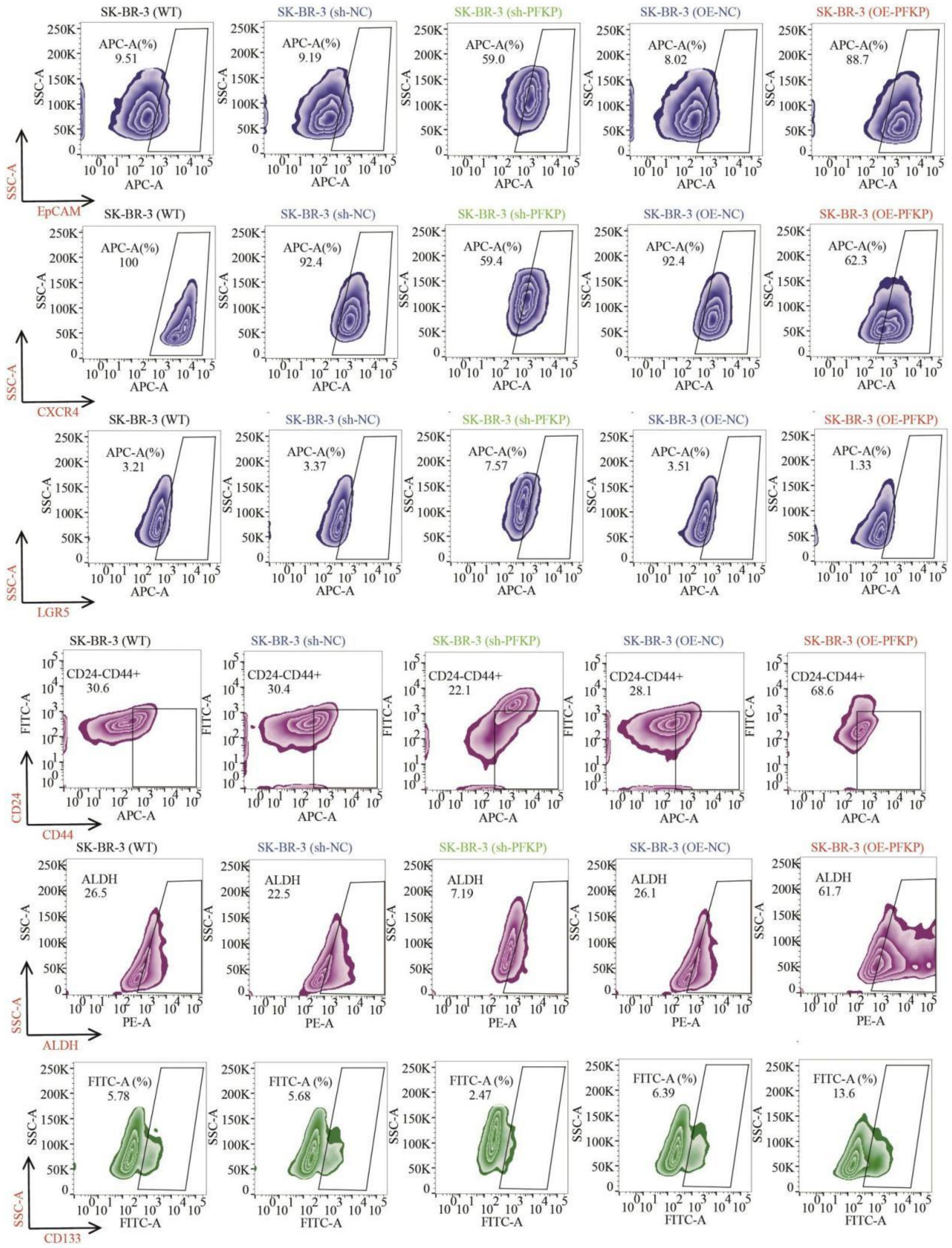

Fig. S5. PFKP promotes the formation of CD133^{+} BCSLCs in SK-BR-3 cells via flow cytometry detection.

In SK-BR-31 cells, the proportion of EpCAM, CXCR4, LGR5, CD24, CD44, CD133, and ALDH positive cells were detected via flow cytometry. Notes: Each experiment was conducted at least four times.

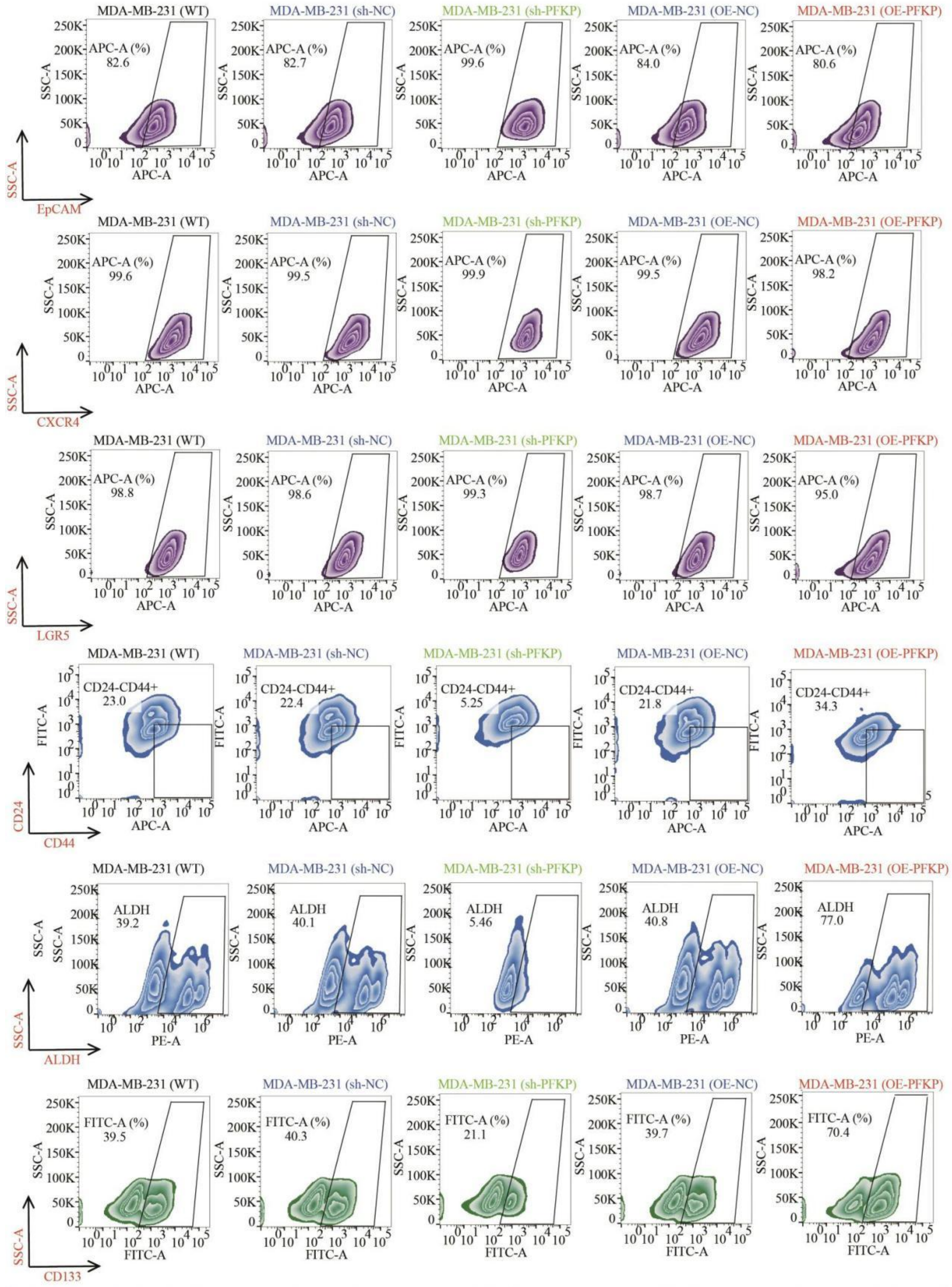

Fig. S6. PFKP promotes the formation of CD133$^+$ BCSLCs in MDA-MB-231 cells via flow cytometry detection.

In MDA-MB-231 cells, the proportion of EpCAM, CXCR4, LGR5, CD24, CD44, CD133, and ALDH positive cells were detected via flow cytometry. Notes: Each experiment was conducted at least four times.

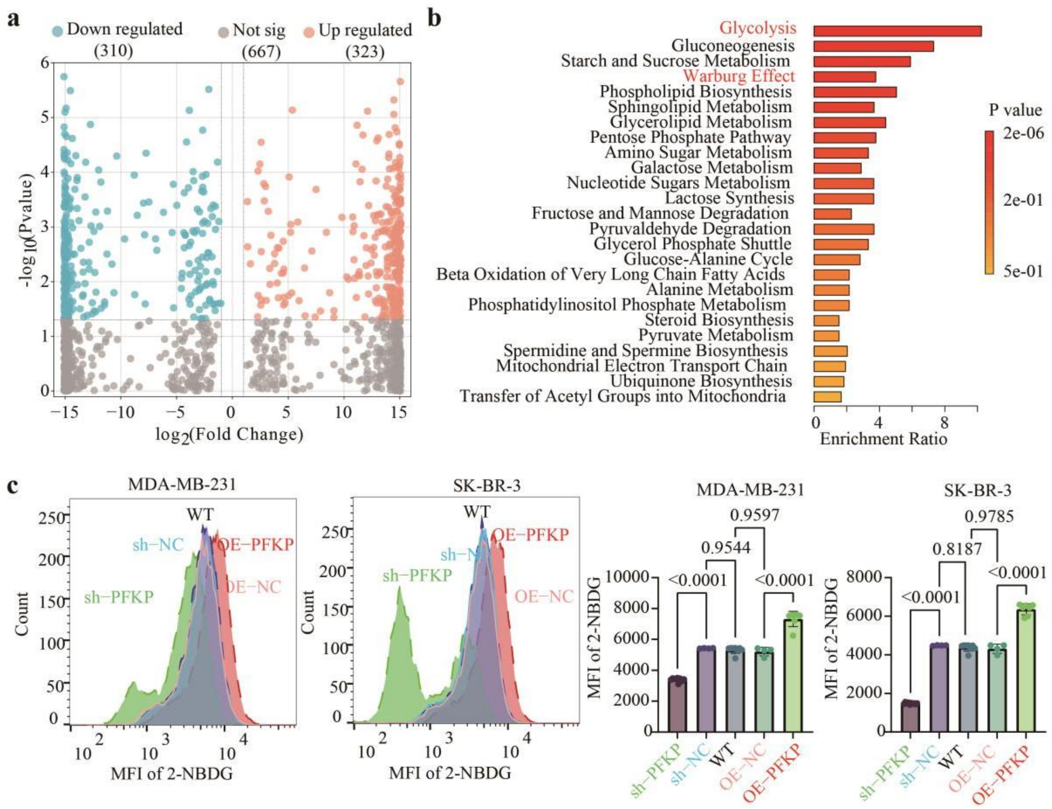

Fig. S7. PFKP regulates glycolysis and glucose uptake in BC cells.

(a) Non-targeted metabolomics was performed to compare metabolic differences between WT and OE-PFKP SK-BR-3 cells. In the OE-PFKP group, 323 metabolites showed a significant decrease and 310 metabolites showed a significant increase compared to the WT group, with selection criteria of |Fold Change (FC)|≥2 and P≤0.05. (b) Functional enrichment analysis was conducted on these 633 differential expressed metabolites (DEMs) using the SMPDB database, revealing a predominant enrichment in the glycolysis pathway. (c) Cells were co-cultured with 5 nM 2-NBDG for 2 hours. The 2-NBDG glucose uptake assay demonstrated that PFKP overexpression enhances glucose uptake in BC cells, while PFKP silencing reduces it.

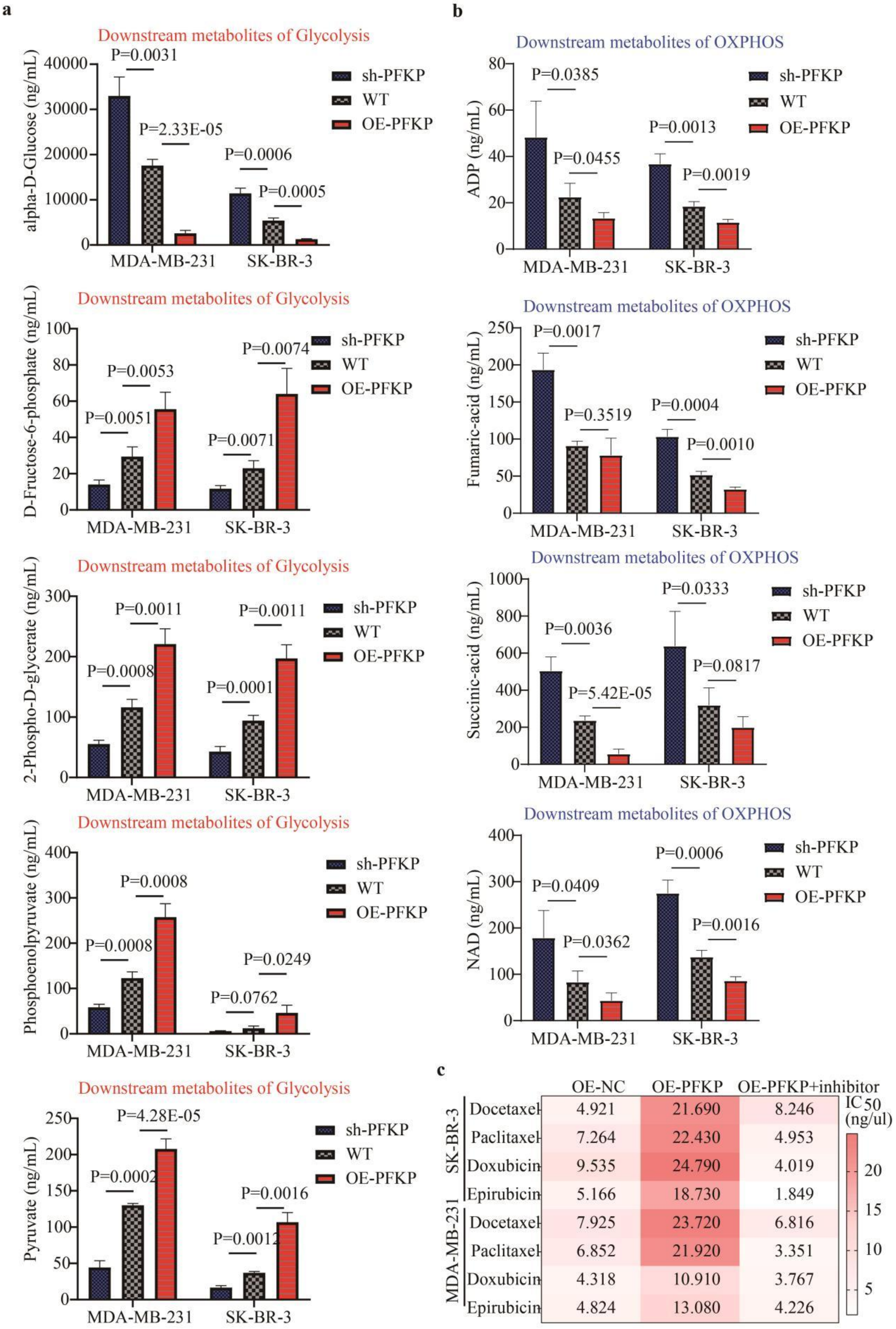

Fig. S8. PFKP regulates glycolysis, oxidative phosphorylation and drug sensitivity in BC cells.

(a) Targeted metabolomics revealed that PFKP overexpression promotes the

levels of glycolysis-related metabolites in BC cells, and silencing PFKP has the opposite effect. (b) Targeted metabolomics also showed that PFKP overexpression inhibits the levels of oxidative phosphorylation-related metabolites in BC cells, with silencing PFKP having the opposite effect. (c) CCK-8 assay results indicate that the addition of 5 uM glycolysis inhibitor for 48 hour can counteract the increase in IC_{50} to TA-based drugs caused by PFKP overexpression in BC cells. Notes: Each experiment was conducted at least four times. Data are presented as mean ± SD.

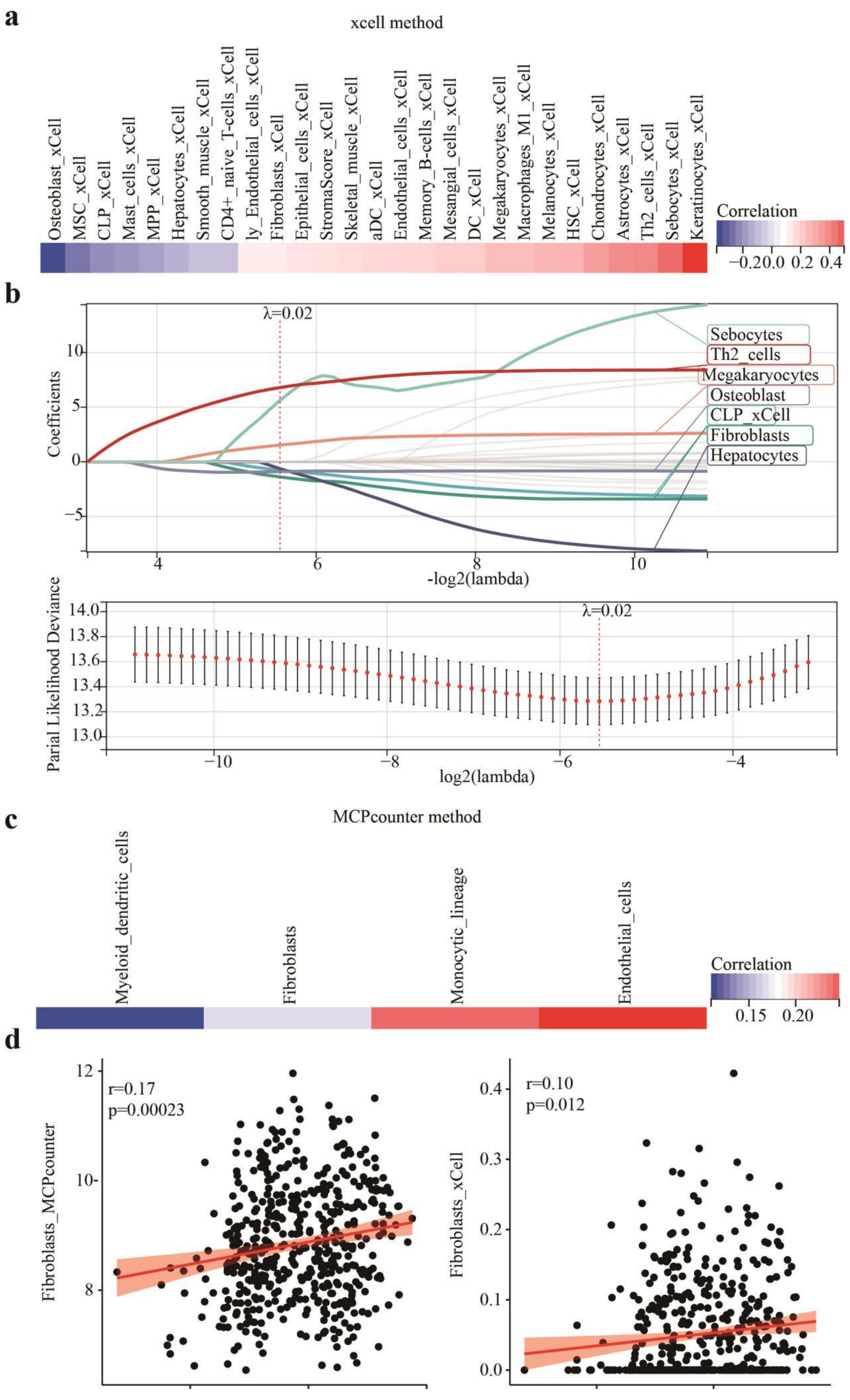

a
xcell method
Osteoblast_xCell
MSC_xCell
CLP_xCell
Mast_cells_xCell
MPP_xCell
Hepatocytes_xCell
Smooth_muscle_xCell
CD4+_naive_T-cells_xCell
ly_Endothelial_cells_xCell
Fibroblasts_xCell
Epithelial_cells_xCell
StromaScore_xCell
Skeletal_muscle_xCell
aDC_xCell
Endothelial_cells_xCell
Memory_B-cells_xCell
Mesangial_cells_xCell
DC_xCell
Megakaryocytes_xCell
Macrophages_M1_xCell
Melanocytes_xCell
HSC_xCell
Chondrocytes_xCell
Astrocytes_xCell
Th2_cells_xCell
Sebocytes_xCell
Keratinocytes_xCell
Correlation
−0.2 0.0 0.2 0.4
b
λ=0.02
Coefficients
-log2(lambda)
Sebocytes
Th2_cells
Megakaryocytes
Osteoblast
CLP_xCell
Fibroblasts
Hepatocytes
Parial Likelihood Deviance
log2(lambda)
c
MCPcounter method
Myeloid_dendritic_cells
Fibroblasts
Monocytic_lineage
Endothelial_cells
Correlation
0.15 0.20
d
r=0.17
p=0.00023
Fibroblasts_MCPcounter
r=0.10
p=0.012
Fibroblasts_xCell

Fig. S9. Correlation of CD133 expression with tumor microenvironment cells in BC.

(a) The 'xCell' algorithm was employed to estimate the composition of TME cells within BC tumors, utilizing data from the GSE25055 and GSE25065 datasets. Subsequently, Pearson’s r method was conducted to identify correlations between CD133 mRNA expression levels and the abundance of TME cells. (b) The lasso-cox analysis was applied to identify TME cells associated with relapse-free survival in the GSE25055 and GSE25065 datasets. (c) The "MCPcounter" method was utilized to calculate TME cells in BC tumors from the GSE25055 and GSE25065 datasets. Pearson’s r method was conducted to evaluate the correlations between CD133 mRNA expression and TME cells. (d) In the GSE25055 and GSE25065 datasets, CD133 mRNA expression was significantly and positively correlated with fibroblasts.

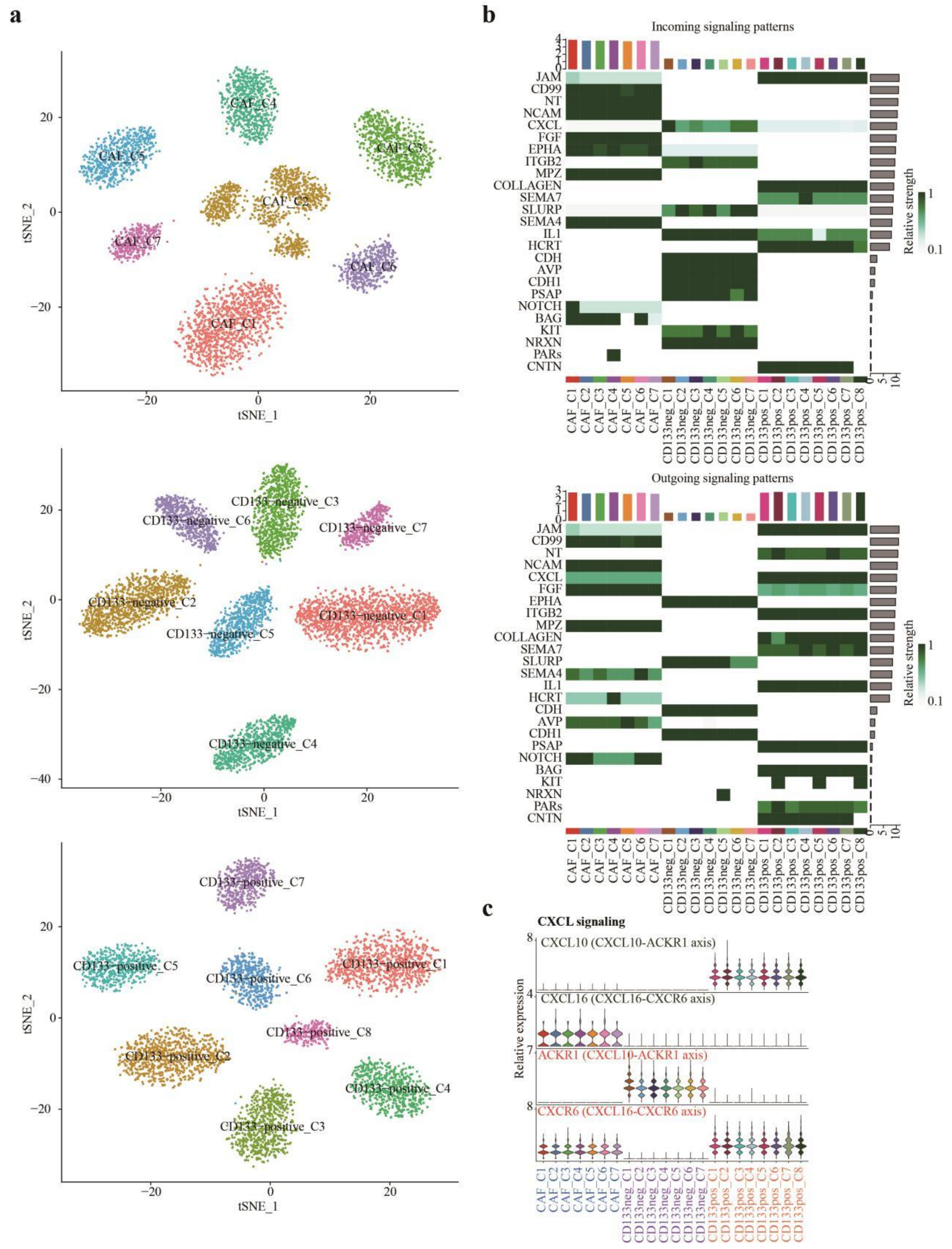

Fig. S10. ScRNA-seq unveils mechanisms of CAF-mediated CD133^{+} BCSLCs formation

(a) Subtype analysis of CAFs, CD133^{-}, and CD133^{+} cells was conducted using the R package "Seurat". (b) Cell communication between CAFs, CD133^{-}, and CD133^{+} cells was calculated using the R package "CellChat". (c) CAFs communicate with CD133^{+} cells via the CXCR6 (CXCL16-CXCR6 axis).

Supplementary Tables

Table. S1. Variations in sensitivity to TA-based chemotherapy between BC patients with basal-type and non-basal-type.

Group	Basal (N=183)	Non-Basal (N=305)	Total (N=488)	P value
Chemosensitivity				8.80E-03
Insensitive	137(28.07%)	192(39.34%)	329(67.42%)	
Sensitive	46(9.43%)	113(23.16%)	159(32.58%)	
Recurrence-free survival				8.10E-07
Recurrence	62(12.70%)	44(9.02%)	106(21.72%)	
Recurrence-free	121(24.80%)	261(53.48%)	382(78.28%)	

Table. S2. The enrolled sequencing profiles in this study.

Datasets	Basal	Non-basal	Sequencing level	Databases	Analytical method	Treatment
GSE25055	58	250	mRNA (microarray)	GEO	Limma	neoadjuvant taxane-anthracycline chemotherapy
GSE25065	74	124	mRNA (microarray)	GEO	Limma	neoadjuvant taxane-anthracycline chemotherapy
TCGA	171	807	mRNA (RNA sequencing)	TCGA	Limma-voom	Unwell defined
Metabric	290	1410	mRNA (microarray)	cbiopotal	Limma	Unwell defined
GSE31448	98	165	mRNA	GEO	Limma	Unwell defined

			(microarray)			
GSE65194	55	98	mRNA (microarray)	GEO	Limma	Unwell defined
GSE87049	14	95	mRNA (microarray)	GEO	Limma	Unwell defined
GSE78958	99	319	mRNA (microarray)	GEO	Limma	Unwell defined
CPTAC	28	88	Protein	cbiopotal	Wilcoxon rank-sum test	Unwell defined

Table. S3. The basic information of enrolled patients.

**Items**		**Basal (n=25)**	**Non-basal (n=40)**
Age (years)		56.81 ± 8.69	59.25 ± 11.37
TNM stage			
	T0	3	5
	T1	6	6
	T2	8	13
	T3	5	7
	T4	3	8
	Tx	0	1
	N0	3	5
	N1	6	18
	N2	7	11
	N3	8	4
	Nx	1	2
	M0	11	25

M1	7	12
Mx	2	3

Table. S4. The primers used in this study.

Gene	Forward (5'-3')	Reverse (5'-3')
GAPDH	GGAGCGAGATCCCTCCAAAAT	GGCTGTTGTCATACTTCTCATGG
PFKP	GCATGGGTATCTACGTGGGG	CTCTGCGATGTTTGAGCCTC
CD133	AGTCGGAAACTGGCAGATAGC	GGTAGTGTTGTACTGGGCCAAT

Table. S5. The antibodies used in this study.

Antibodies	Cat No.	Company	Experiments	Dilution	Reactivity
GAPDH	60004-1-Ig	proteintech	WB	1:1500	Rabbit Anti-Human
Beta-actin	GTX109639-S	genetex	WB	1:1500	Rabbit Anti-Human
PFKP	13389-1-AP	proteintech	WB	1:1500	Rabbit Anti-Human
CD133	A12711	abclonal	WB	1:1000	Goat Anti-Rabbit
HRP-IgG	SA00001-2	proteintech	WB	1:2000	Goat Anti-Rabbit

Supplementary Materials for WB

Fig 1f

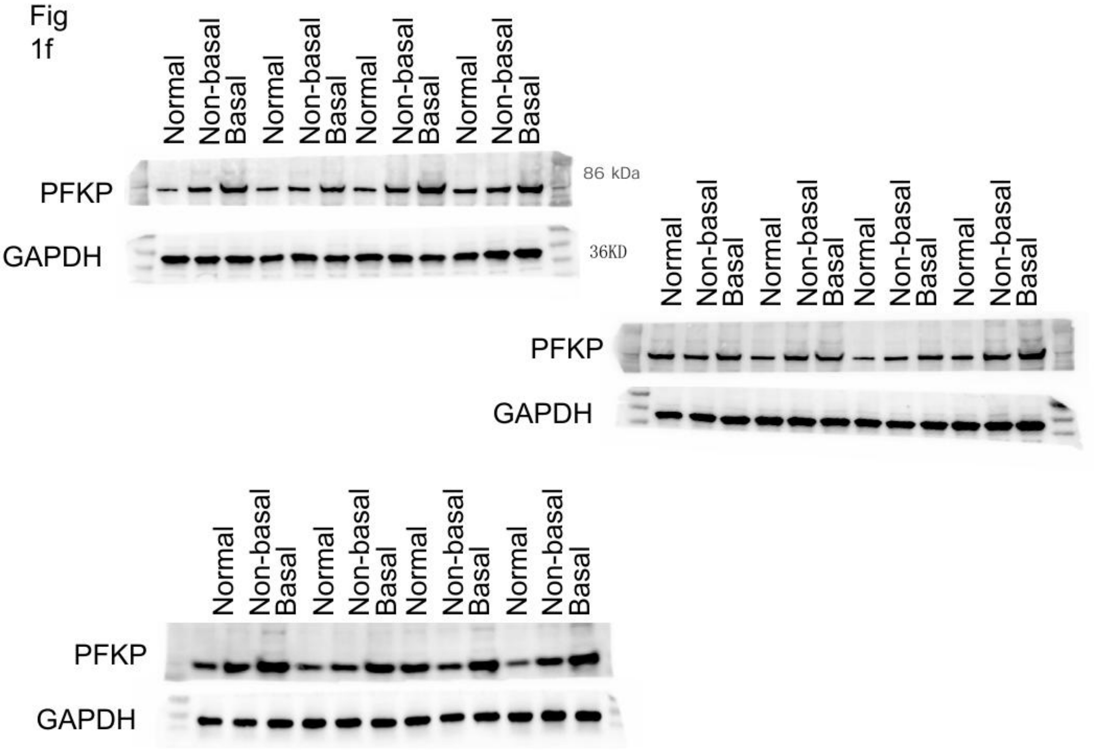
Normal
Non-basal
Basal
PFKP
GAPDH
86 kDa
36KD

Fig 2a

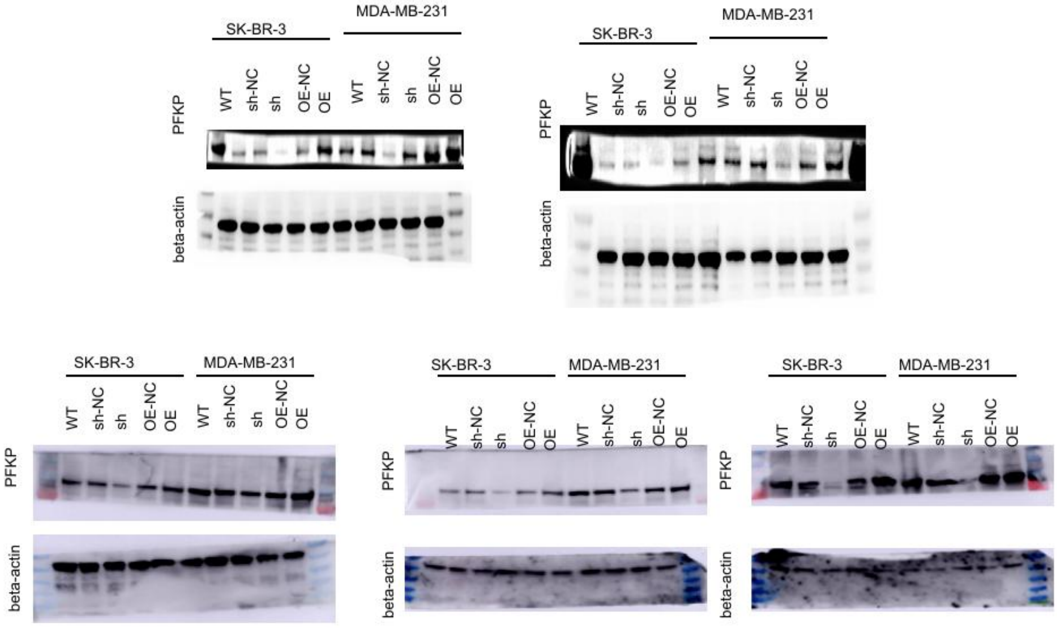
SK-BR-3
MDA-MB-231
WT
sh-NC
sh
OE-NC
OE
PFKP
beta-actin

Fig
2h

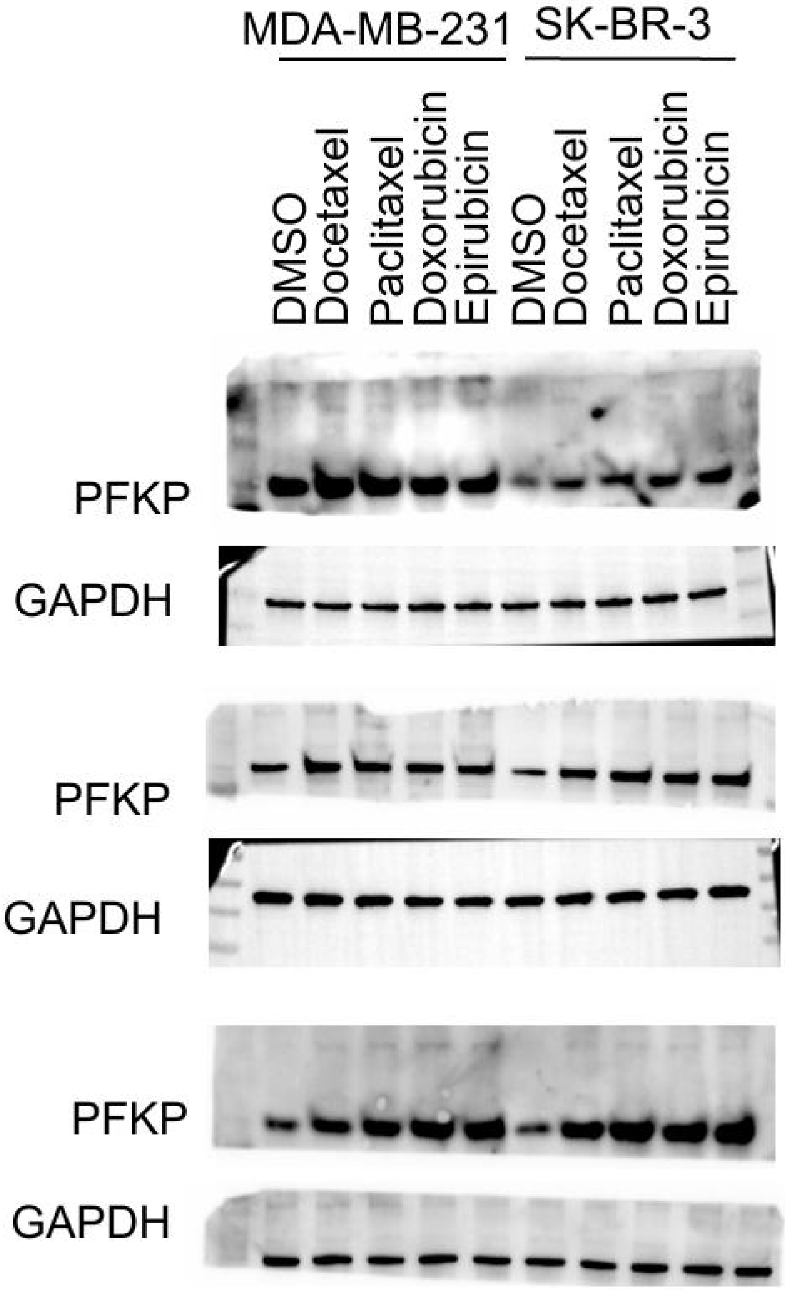
MDA-MB-231
SK-BR-3
DMSO
Docetaxel
Paclitaxel
Doxorubicin
Epirubicin
DMSO
Docetaxel
Paclitaxel
Doxorubicin
Epirubicin
PFKP
GAPDH
PFKP
GAPDH
PFKP
GAPDH

Fig
6g

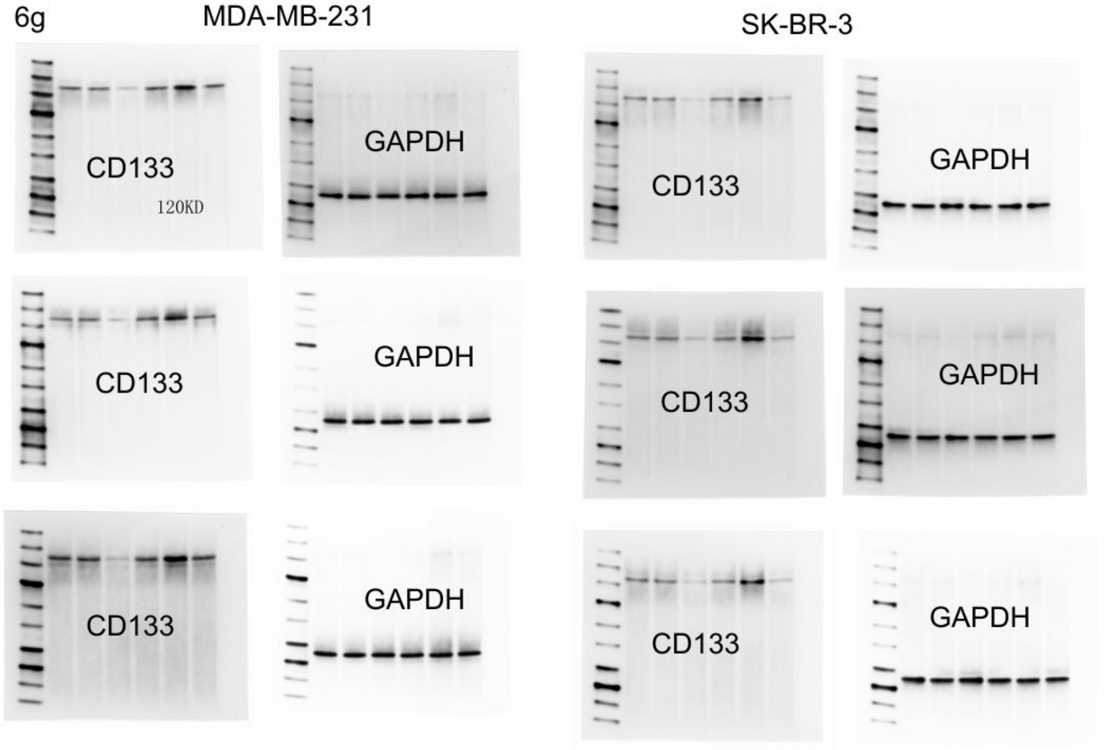
MDA-MB-231
SK-BR-3
CD133
120KD
GAPDH
CD133
GAPDH
CD133
GAPDH
CD133
GAPDH
CD133
GAPDH
CD133
GAPDH
